# Role of the *Escherichia coli* ubiquinone-synthesizing UbiUVT pathway in adaptation to changing respiratory conditions

**DOI:** 10.1128/mbio.03298-22

**Published:** 2023-06-07

**Authors:** Rodrigo Arias-Cartin, Katayoun Kazemzadeh Ferizhendi, Emmanuel Séchet, Ludovic Pelosi, Corinne Loeuillet, Fabien Pierrel, Frédéric Barras, Emmanuelle Bouveret

**Affiliations:** 1 Département de Microbiologie, Institut Pasteur, Université Paris-Cité, UMR CNRS 6047, SAMe Unit, Paris, France; 2 Univ. Grenoble Alpes, CNRS, UMR 5525, VetAgro Sup, Grenoble INP, TIMC, Grenoble, France; 3 Univ. Grenoble Alpes, INSERM U1209, CNRS UMR 5309, Institute for Advanced Biosciences, Team Genetics Epigenetics and Therapies of Infertility, Grenoble, France; University of California, Irvine, California, USA

**Keywords:** quinone, *E. coli*, Fnr, respiration, UbiTUV

## Abstract

Isoprenoid quinones are essential for cellular physiology. They act as electron and proton shuttles in respiratory chains and various biological processes. *Escherichia coli* and many α-, β-, and γ-proteobacteria possess two types of isoprenoid quinones: ubiquinone (UQ) is mainly used under aerobiosis, while demethylmenaquinones (DMK) are mostly used under anaerobiosis. Yet, we recently established the existence of an anaerobic O_2_-independent UQ biosynthesis pathway controlled by *ubiT*, *ubiU*, and *ubiV* genes. Here, we characterize the regulation of *ubiTUV* genes in *E. coli*. We show that the three genes are transcribed as two divergent operons that are both under the control of the O_2_-sensing Fnr transcriptional regulator. Phenotypic analyses using a *menA* mutant devoid of DMK revealed that UbiUV-dependent UQ synthesis is essential for nitrate respiration and uracil biosynthesis under anaerobiosis, while it contributes, though modestly, to bacterial multiplication in the mouse gut. Moreover, we showed by genetic study and ^18^O_2_ labeling that UbiUV contributes to the hydroxylation of ubiquinone precursors through a unique O_2_-independent process. Last, we report the crucial role of *ubiT* in allowing *E. coli* to shift efficiently from anaerobic to aerobic conditions. Overall, this study uncovers a new facet of the strategy used by *E. coli* to adjust its metabolism on changing O_2_ levels and respiratory conditions. This work links respiratory mechanisms to phenotypic adaptation, a major driver in the capacity of *E. coli* to multiply in gut microbiota and of facultative anaerobic pathogens to multiply in their host.

**IMPORTANCE:**

Enterobacteria multiplication in the gastrointestinal tract is linked to microaerobic respiration and associated with various inflammatory bowel diseases. Our study focuses on the biosynthesis of ubiquinone, a key player in respiratory chains, under anaerobiosis. The importance of this study stems from the fact that UQ usage was for long considered to be restricted to aerobic conditions. Here we investigated the molecular mechanism allowing UQ synthesis in the absence of O_2_ and searched for the anaerobic processes that UQ is fueling in such conditions. We found that UQ biosynthesis involves anaerobic hydroxylases, that is, enzymes able to insert an O atom in the absence of O_2_. We also found that anaerobically synthesized UQ can be used for respiration on nitrate and the synthesis of pyrimidine. Our findings are likely to be applicable to most facultative anaerobes, which count many pathogens (*Salmonella*, *Shigella*, and *Vibrio*) and will help in unraveling microbiota dynamics.

## INTRODUCTION

Isoprenoid quinones are widely distributed in the three domains of life and globally act as electron and proton carriers (1). They serve in many processes of bacterial physiology and electron transport chains like photosynthesis, for example, plastoquinone and phylloquinone, and respiration, for example, UQ and menaquinone (MK) (2). Isoprenoid quinones are composed of a quinone ring and a polyisoprenoid side chain whose length varies between organisms (for instance, UQ_8_ in *E. coli* and UQ_9_ in *Pseudomonas aeruginosa*). Many proteobacteria, such as *E. coli*, produce two main types of quinones: benzoquinones, represented by UQ, and naphthoquinones, such as MK and demethylmenaquinone (DMK). In respiratory chains, quinones transfer electrons from primary dehydrogenases to terminal reductases. For decades, *E. coli* aerobic and anaerobic respiratory chains were thought to rely on UQ and MK/DMK, respectively. Yet, we have recently discovered a new pathway for UQ biosynthesis under anaerobiosis, opening the way to a more complex and redundant model for bacterial respiratory metabolism (3).

Aerobic UQ biosynthesis pathway includes nine steps (4) (Fig. S1). It begins with the conversion of chorismate to 4-hydroxybenzoate (4HB) by the chorismate lyase UbiC. Then, the phenyl ring of the 4HB precursor undergoes condensation with a 40-carbon-long isoprenoid chain in a reaction catalyzed by the UbiA enzyme. Subsequently, a series of modifications on the 4HB ring by two methylases (UbiE and UbiG), a two-component decarboxylase (UbiD, UbiX), and three hydroxylases (UbiI, UbiH, and UbiF) generate the final UQ_8_ product. The flavin adenine dinucleotide monooxygenases UbiI, UbiH, and UbiF use molecular O_2_ for their hydroxylation reaction (5 - 7). An atypical kinase-like protein called UbiB is also involved in UQ_8_ synthesis, but its exact role remains elusive (8). In addition, two non-enzymatic factors are required, UbiJ and UbiK, which may allow UbiIEFGH enzymes to assemble in a cytoplasmic 1 MDa complex, referred to as the Ubi metabolon (9). Also, UbiJ and UbiK bind lipids, which may help the hydrophobic UQ biosynthesis to proceed inside a hydrophilic environment.

Anaerobic UQ biosynthesis is formed by a subset of the enzymes of the aerobic pathway, namely UbiA, UbiB, UbiC, UbiD, UbiE, UbiG, and UbiX, that function with UbiT, UbiU, and UbiV proteins solely required under anaerobiosis (3) (Fig. S1). Like its homolog counterpart UbiJ, UbiT contains an SCP2 lipid–binding domain. Strikingly, UbiU and UbiV do not exhibit any sequence similarity or functional relatedness with the hydroxylases UbiI, UbiH, or UbiF. UbiU and UbiV each contain an iron–sulfur ([4Fe–4S]) cluster coordinated by four conserved cysteine residues embedded in the so-called protease U32 domain, and they form a soluble UbiUV complex (3). Interestingly, two other members of the U32 protein family, RlhA and TrhP, are involved in hydroxylation reactions. They introduce specific nucleotide modifications, respectively, in the 23S rRNA or in some tRNAs (10 - 12).

In this work, we aimed at identifying the conditions under which UbiUVT proteins are produced and the genetic regulatory mechanisms involved, and the physiological role of UbiUVT. We concluded that (i) thanks to Fnr control, UbiUV ensures the production of UQ under a range of O_2_ levels, from anaerobiosis to microaerobiosis, (ii) a dual anaerobic/aerobic regulation allows UbiT to secure a rapid shift from anaerobic UbiUV-dependent UQ synthesis to an aerobic UbiIHF-dependent UQ synthesis, and (iii) UbiUV-synthesized UQ can be used for nitrate respiration and anaerobic pyrimidine biosynthesis. We also showed that UbiUV acts as O_2_-independent hydroxylases paving the way for future studies toward the characterization of a new type of chemistry.

## MATERIALS AND METHODS

### Strain constructions

Most knockout strains were obtained by generalized Φ P1 transduction using donor strains from the Keio collection (13). For introducing the sequential peptide affinity (SPA) tag on the chromosome or for the generation of specific knockouts, PCR recombination with the lambdaRed system was used, using the oligonucleotides indicated in Table 1 (14, 15). When necessary, the antibiotic resistance marker was removed using FLP recombinase expression from plasmid pCP20 as described previously (16). Cassette removal and plasmid loss were verified by antibiotic sensitivity and confirmed by PCR amplification. Point mutations were introduced on the chromosome using the pKO3 vector (17).

**TABLE 1:** Strains used in this study

Strain	Genotype	Construction	Reference
FBE051	MG1655		Lab strain
FBE229	∆*ubiUV::kan*	PCR LL792/LL715 on pKD4, recombined in BW25113, followed by Φ P1 transduction in MG1655	This work
FBE230	∆*ubiUV::cat*	PCR LL792/LL715 on pKD3, recombined in BW25113, followed by Φ P1 transduction in MG1655	This work
FBE254	∆*ubiT*::kan		(3)
FBE255	∆*ubiT*::cat		(3)
FBE354	∆*fnr*::*aadA*		(18)
FBE430	∆*menA*::kan	Φ P1 transduction from Keio ∆*menA* to MG1655	This work
FBE501	∆*menA*	Kanamycin cassette removed from FBE430 with pCP20	This work
FBE526	∆*menA* ∆ubi*T*::kan	Φ P1 transduction from FBE254 to FBE501	This work
FBE527	∆*menA* ∆*ubiUV*::kan	Φ P1 transduction from FBE229 to FBE501	This work
FBE947	∆*ubiH*::Kan ∆*ubiUV*::cat	Φ P1 transduction from FBE230 to FBE253	This work
FBE1013	∆*menA* ∆*ubiH*::kan	Φ P1 transduction from FBE253 to FBE501	This work
FBE1032	∆*pyrD*::kan	Φ P1 transduction from Keio ∆*pyrD* to MG1655	This work
FBE253	∆*ubiH*::kan		(19)
FBE510	∆*ubiD*::cat		(3)
FBE512	∆*ubiF::*kan		(6)
FBE515	∆*ubiK*::kan		(20)
FBE518	∆*ubiA*::cat		(3)
FBE690	∆*ubiI*	Φ P1 transduction from Keio ∆*ubiI* to MG1655 then kanamycin cassette removed with pCP20	This work
FBE713	∆*ubiIK*	Φ P1 transduction from FBE515 to FBE690	This work
FBE576	*∆ubiIF∆ubiHF::cat*		(19)
FBE650	∆*ubiIHF*	FBE576 strain cured with pCP20 plasmid	This work
FBE514	∆*ubiJ::cat*		(21)
FBE794	∆*ubiJ*∆*ubiH*	Φ P1 transduction from FBE514 to FBE253	This work
FBE264	∆*ubiJ*∆*ubiF*	Φ P1 transduction from FBE514 to FBE512	This work
FBE795	∆*ubiT*∆*ubiH*	Φ P1 transduction from FBE255 to FBE253	This work
FBE265	∆*ubiT*∆*ubiF*	Φ P1 transduction from FBE255 to FBE512	This work
FBE792	∆*ubiH*∆*ubiA*	Φ P1 transduction from FBE518 to FBE253	This work
FBE793	∆*ubiH*∆*ubiD*	Φ P1 transduction from FBE510 to FBE253	This work
FBE656	*ubiU*-SPA-kan	Φ P1 transduction DY330ubiU-SPA in MG1655	This work
FBE789	*ubiV*-SPA-kan	Φ P1 transduction DY330ubiV-SPA in MG1655	This work
FBE655	*ubiT*-SPA-kan	Recombination PCR LL710/LL711 on pJL148 in BW25113 then Φ P1 transduction in MG1655	This work
FBE695	∆*fnr ubiU*-SPA-kan	Φ P1 transduction from FBE656 to FBE354	This work
FBE696	∆*fnr ubiV*-SPA-kan	Φ P1 transduction from FBE789 to FBE354	This work
FBE694	∆*fnr ubiT*-SPA-kan	Φ P1 transduction from FBE655 to FBE354	This work
FBE882	*ubiT-SPA* mutF1	Recombination pKO3-*ubiTU*mutF1 (pVP222) in FBE655	This work
FBE883	*ubiT*-SPA mut∆F2	Recombination pKO3-*ubiTU*mut∆F2 (pVP223) in FBE655	This work
FBE884	*ubiV*-SPA mutF1	Recombination pKO3-*ubiTU*mutF1 (pVP222) in FBE789	This work
FBE885	*ubiV-SPA* mut∆F2	Recombination pKO3-*ubiTU*mut∆F2 (pVP223) in FBE789	This work
FBE855	*yhbS-SPA-kan*	Recombination PCR ebp292/293 on pJL148 in BW25113 then Φ P1 transduction in MG1655	This work
FBE856	*∆fnr yhbS-SPA-kan*	Φ P1 transduction from FBE856 to FBE354	This work
FBE857	*∆yhbS*	Φ P1 transduction from Keio ∆*yhbS* to MG1655	This work
FBE858	*∆yhbS∆menA*	Φ P1 transduction from Keio ∆*yhbS* to FBE354	This work
FBE484	MP7	Lambda att : tetR tetA-mCherry	(22)
FBE485	MP13	Lambda att : tetR tetA-gfpmut3.1	(22)
FBE550	*MP13∆ubiUV*	Φ P1 transduction from FBE229 to FBE485	This work
FBE888	*MP13∆menA*	Φ P1 transduction from FBE430 to FBE485	This work

For mouse intestine colonization experiments, we used MP7 and MP13 strains, which derive from the commensal *E. coli* MP1 strain (22). MP7 and MP13 express, respectively, mCherry or green fluorescent protein (GFP) under the control of a tetracycline-inducible promoter. ∆*menA* and ∆*ubiUV* deletions were introduced in MP13 using generalized Φ P1 transduction.

### Plasmid constructions

pUA66 and pUA-*ubiUV*p plasmids were obtained from the library of *E. coli* promoters fused to the GFP coding sequence (23). The *ubiT* transcriptional fusions were constructed using primers indicated in Table 3 and cloned in XhoI/BamHI sites of pUA66. Expression plasmids for *ubiUV* and *fnr* were constructed using primers indicated in Table 3 and cloned in EcoRI/SalI sites of the pBAD24 vector (24). Expression plasmids for *ubiIHF* and *ubiM_Neisseria* genes were constructed using primers indicated in Table 3 and cloned in EcoRI/XhoI sites of pTet vector. A region of 1,275 base pairs encompassing *ubiU* and *ubiT* promoters was cloned in pKO3 vector (17). Mutations were introduced in the pKO3-*ubiTU* vector, in the pBAD-*ubiUV*, and in the transcriptional fusions by PCR mutagenesis on a plasmid, using the oligonucleotides indicated (Table 2 and 3).

**TABLE 2:** Plasmids used in this study

Plasmid	Name	Description/construction	Source
pCP20	pCP20	Ap, Cm, FLP recombinase expression	(16)
pEB227	pBAD24	AmpR—ColE1 ori—PBAD promoter	(24)
pEB267	pKD46	AmpR—ts ori—lambda Red genes	(15)
pEB268	pKD3	AmpR—FRT-cat-FRT	(15)
pEB269	pKD4	AmpR—FRT-kanaR-FRT	(15)
pEB794	pJL148	AmpR—SPA-FRT-kanaR-FRT	(14)
pES154	pBAD-*ubiUV*	PCR on MG1655 genomic DNA ebp134/136 (EcoRI/XhoI) in pBAD24 (EcoRI/SalI)	This work
pES185	pBAD-*ubiU*(C176A)*V*	Mutagenesis PCR ebp178/179 on pES154	This work
pES184	pBAD-ubiUV-SPA	PCR ebp134/ebm968 on strain FBE696 (EcoRI/XhoI) in pBAD24 (EcoRI/SalI)	This work
pVP040	pBAD-*fnr*	PCR on MG1655 genomic DNA ebp31/32 (MfeI/XhoI) in pBAD24 (EcoRI/SalI)	This work
pEB1242	pASK-IBA37plus	AmpR—ColE1 ori—TetR promoter—6His	IBA
pEB1823	pTet	PCR mutagenesis ebm1567/1568 on pEB1242 to remove 6His tag	This work
pES060	pTet-*ubiI*	PCR on MG1655 genomic DNA ebp64/65 (EcoRI/XhoI) in pTet (EcoRI/XhoI)	This work
pES059	pTet-*ubiH*	PCR on MG1655 genomic DNA ebp61/62 (EcoRI/XhoI) in pTet (EcoRI/XhoI)	This work
pES143	pTet-*ubiF*	PCR on MG1655 genomic DNA ebp37/38 (EcoRI/XhoI) in pTet (EcoRI/XhoI)	This work
pES151	pTet-*ubiM*_*Neisseria*	PCR on MG1655 genomic DNA ebp139/140 (EcoRI/XhoI) in pTet (EcoRI/XhoI)	This work
pEB898	pUA66	KanR—pSC101 ori—GFPmut2	(23)
	pUA-*ubiUVp*		(23)
pVP220	pUA-*ubiUVp*mutF1	Mutagenesis PCR Ebp287/288 on pUA-*ubiU*	This work
	pUA-*ubiT*p1p2		(23)
pVP169	pUA-*ubiTp1*	PCR on MG1655 genomic DNA ebp191/192 (XhoI/BamHI) in pUA66 (XhoI/BamHI)	This work
pVP170	pUA-*ubiTp2*	PCR on MG1655 genomic DNA ebp193/194 (XhoI/BamHI) in pUA66 (XhoI/BamHI)	This work
pVP187	pUA-ubiTp1p2∆F2	Mutagenesis PCR Ebp237/238 on pUA-*ubiT*	This work
pVP221	pUA-ubiTp1p2mutF2	Mutagenesis PCR Ebp289/290 on pUA-*ubiT*	This work
pEB232	pKO3	camR, pSC101 ori, *sacB*	(17)
pVP219	pKO3-*ubiTU*	PCR on MG1655 genomic DNA ebp276/291 (XhoI/BamHI) in pKO3 (SalI/BamHI)	This work
pVP222	pKO3-*ubiTU*mutF1	Mutagenesis PCR ebp287/288 on pVP219	This work
pVP223	pKO3-*ubiTUmut*∆F2	Mutagenesis PCR ebp237/238 on pVP219	This work

**TABLE 3:** Primers used in this study

Primers	Sequence	Use
ebm968	ttgctcgagAAGCAGCTCCAGCCTACACG	*ubiV*-SPA RV
ebm1567	GAAATAATTTTGTTTAACTTTAAGAAGGAGATGAATTCGAGCTCGGTACCCG	pEB1823
ebm1568	CGGGTACCGAGCTCGAATTCATCTCCTTCTTAAAGTTAAACAAAATTATTTC	pEB1823
Ebp31	GAGCAATTGatgATCCCGGAAAAGCGAATTATAC	*fnr* FW MfeI
Ebp32	acgctcgagtcaGGCAACGTTACGCGTATG	*fnr* RV XhoI
Ebp37	actgaattcatgACAAATCAACCAACGGAAATTG	*ubiF* FW EcoRI
Ebp38	acgctcgagctaCAACCCTAACGCATATTTCAGC	*ubiF* RV XhoI
Ebp61	actgaattcATGAGCGTAATCATCGTCGGTG	*ubiH* FW EcoRI
Ebp62	acgctcgagTtAACGCGCCACCCAACC	*ubiH* RV XhoI
Ebp64	actgaattcATGCAAAGTGTTGATGTAGCCATTG	*ubiI* FW EcoRI
Ebp65	acgctcgagTTAACGCAGCCATTCAGGCAAATC	*ubiI* RV XhoI
Ebp134	actgaattcatgGAGCTGCTCTGCCCTG	*ubiU* FW EcoRI
Ebp136	actgaattcATGAAATATTCCTTAGGGCCAGTG	*ubiV* RV XhoI
Ebp139	actgaattcATGGGTTTTGATATAATCGCCTATC	*ubiM* FW EcoRI
Ebp140	acgctcgagTCAACCGGTCAGTTGTTTGGTAATC	*ubiM* RV XhoI
Ebp178	TTATGTCGGAAGGTCGTgcCTATCTGTCGTCGTATC	*ubiU*(C179A) FW
Ebp179	GATACGACGACAGATAGgcACGACCTTCCGACATAA	*ubiU*(C179A) RV
Ebp191	acgctcgagTTAAGCGCCGGGAGATTTCC	*ubiTp1 FW*
Ebp192	cgggatccTGCTGCTACCACCAATACAAC	*ubiTp1 RV*
Ebp193	cgggatccTTTTAGCGCAAATGGCGTCAG	*ubiTp2 RV*
Ebp194	acgctcgagAGCAGCAATTTCATATGGAATTGTTG	*ubiTp2 FW*
Ebp237	ttggtggtagcagcaatttcatatggaattgctatgttatttttctgat	mut∆Fnr2 FW
Ebp238	atcagaaaaataacatagcaattccatatgaaattgctgctaccaccaa	mut∆Fnr2 RV
Ebp275	actgaattcaTGTTGGATAAACTGCGTTCCC	*ubiT* FW
Ebp276	acgctcgagTTAGCATGGTTCACCTACCGATG	*ubiT* RV XhoI
Ebp285	acgctcgagTTAAAAGCGATTGAAATGCTCG	*yhbS* RV
Ebp287	CAACTTTAACTGCCTTAAtcatcAAATTGTCGCAGCAAG	mutFnr1 FW
Ebp288	CTTGCTGCGACAATTTgatgaTTAAGGCAGTTAAAGTTG	mutFnr1 RV
Ebp289	CAGCAATTTCATATGGAATTGcatgaTTATACCGCTATGTTATTTTTC	mutFnr2 FW
Ebp290	GAAAAATAACATAGCGGTATAAtcatgCAATTCCATATGAAATTGCTG	mutFnr2 RV
Ebp291	cgggatccTACGACGACAGATAGCAACGAC	*ubiU* RV BamHI
Ebp292	GGCGTTACCGGCCTGGTTGAGTATCACGAGCATTTCAATCGCTTTTCCATGGAAAAGAGAAG	yhbS-tag FW
Ebp293	GCGCAGGGTTTGCAGAGCTGTTAAGCAGTCTGCAAACCCCGGAGACATATGAATATCCTCCTTAG	yhbS-tagRV
LL710	AAAACCGCGCCTGAAACCAAACAGACATCGGTAGGTGAACCATGCTCCATGGAAAAGAGAAG	ubiT-tag FW
LL711	GCAGGGCATCAATACCCGGCGCATCAATGGGAATTTCTACTCGAACATATGAATATCCTCCTTAG	ubiT-tagRV
LL715	aaagagtagttaaagttgttaacaaagtgagctatttacCATATGAATATCCTCCTTA	RV ubiV Wanner
LL792	catttttgcgttttgatagcgcaaccttcaggaaaaattGTGTAGGCTGGAGCTGCTTC	FW ubiU Wanner

### Media and growth conditions

Strains were grown in LB Miller (10 g/L of tryptone, 10 g/L of NaCl, and 5 g/L of yeast extract) or M9 medium (6 g/L Na_2_HPO_4_•7H_2_O, 3 g/L KH_2_PO_4_, 0.5 g/L NaCl, 1 g NH_4_Cl, 2 mM MgSO_4_, 1 mg/mL thiamine) supplemented with 0.2% glucose, 0.2% glycerol, or 50 mM succinate as the carbon source. For anaerobic cultures, media were degassed and incubated in anaerobic environment for at least 24 hours prior to use, if necessary supplemented with 25 mM KNO_3_ as electron acceptor and uracil 25 μg/mL or casamino acids at 0.05%.

For microaerobic experiments, media and plates were pre-equilibrated and cells were cultured in a Whitley H35 hypoxic station with 95% N_2_, 5% CO_2_, and the desired O_2_ concentration. Humidity and temperature were set up at 85% and 37°C, respectively. For anaerobic–aerobic shift experiments, all anaerobic steps were performed in a JACOMEX Campus anaerobic chamber under N_2_ atmosphere at 1 ppm O_2_ maximum. Cells were first incubated anaerobically in LB agar plates supplemented with 0.2% glucose overnight at 37°C. The next day, cells were cultured anaerobically in 3-mL LB supplemented with 25 mM NO_3_^−^ for 24 hours at 37°C. Still under anaerobiosis, cells were collected by centrifugation, supernatant was discarded, and pellets were washed twice using 1 mL of M9 medium without carbon source and normalized at 0.1 OD units in M9 medium supplemented with 50 mM sodium succinate. At this point, cultures were moved out to atmospheric air, and growth was followed by triplicate at 37°C on 200 µL of culture in a 96-well plate using a TECAN infinite M200 plate reader. At 40 hours of culture, cells were diluted 1/20 in a new M9 50 mM sodium succinate medium and readings were resumed until 60 hours.

#### Aerobic and anaerobic cultures for quinone analysis

For aerobic cultures, 5 mL of LB medium, supplemented with ampicillin (100 µg/mL) and 0.05% arabinose when necessary to induce the expression from the pBAD vectors, was inoculated with 100 µL of overnight culture in glass tubes (15 cm long and 2 cm in diameter) and incubated at 37°C, 180 rpm overnight.

Anaerobic cultures were performed in Hungate tubes as previously described (3). Briefly, LB medium was supplemented with 100 mM KNO_3_ as the final electron acceptor, 100 mg/L L-cysteine (adjusted to pH 6 with NaOH) to reduce residual molecular oxygen, and 2.5 mg/L reasazurin. This medium was distributed in Hungate tubes and deoxygenated by high-purity argon bubbling for 40 minutes. The Hungate tubes were sealed and autoclaved. The resazurin was initially purple, it turned to pink after deoxygenation and become colorless after autoclave. The preculture was performed overnight at 37°C in Eppendorf tubes filled to the top with LB medium containing 100 mM KNO_3_. The Hungate tubes were then inoculated through the septum with disposable syringes and needles with 100 µL of precultures and incubated at 37°C without agitation. The resazurin remained colorless during culture indicating anaerobic conditions.

For anaerobic to aerobic shift assay, MG1655 WT, Δ*ubiUV*, and Δ*ubiT* strains were grown anaerobically in Hungate tubes for ~4 hours. Then, 26 µL of chloramphenicol (200 µg/mL) was injected through the septum with a Hamilton syringe. After 20 minutes, the Hungate tubes were unsealed and 2 mL of cultures was taken for lipid extraction and quinone analysis. The rest of cultures was transferred to 250-mL Erlenmeyer flasks and placed at 37°C and 180 rpm for 2 hours. Two-milliliter aliquots of cultures were taken at 30 minutes and 120 minutes after the transition to ambient air for lipid extraction and quinone analysis.

### SDS-PAGE and western blotting

Total cell extracts were prepared by resuspending cell pellets in Laemli buffer 1× at a concentration of 0.3 optical density at 600 nm (OD_600 nm_) units in 10 µL, and then heating for 10 minutes at 95°C. After the separation of 8 µL of total cell extracts on SDS-PAGE, electrotransfer onto nitrocellulose membranes was performed using Trans-Blot turbo transfer system from Bio-Rad. After blocking in phosphate-buffered saline (PBS) 1× + milk 5%, SPA-tagged proteins were detected with monoclonal anti-Flag M2 antibody purchased from Sigma. YbgF protein was used as an internal control and revealed with polyclonal anti-YbgF antibodies. Fluorescent secondary antibodies were, respectively, IRDye 800 anti-mouse and IRDye 680 anti-rabbit purchased from Li-Cor. Scanning and quantification were performed on a Li-Cor Odyssey-Fc imaging system, reading at 700 nm (for YbgF detection) or 800 nm (for Flag detection).

### Transcriptional fusions with GFP

We used several clones from the *E. coli* transcriptional fusions library (23) and we constructed the required additional transcriptional fusions (see above for plasmid construction and Table 2). ∆*fnr E. coli* strain was co-transformed with plasmids carrying the *gfp* transcriptional fusions and compatible pBAD24 or pBAD-*fnr* plasmids. Selection plates were incubated at 37°C for 16 hours. Six hundred microliters of LB medium supplemented with kanamycin and ampicillin, and with 0.02% arabinose for pBAD-driven expression, were incubated (four biological replicates for each assay) and grown for 16 hours at 37°C in 96-well polypropylene plates of 2.2-mL wells in anaerobiosis. Cells were pelleted and resuspended in PBS supplemented with 30 µg/mL chloramphenicol and incubated at 4°C for 1 hour before fluorescent intensity measurement was performed in a TECAN infinite M200 plate reader. One hundred fifty microliters of each well was transferred into a black Greiner 96-well plate for reading OD_600 nm_ and fluorescence (excitation: 485 nm; emission: 530 nm). The expression levels were calculated by dividing the intensity of fluorescence by OD_600 nm_, after subtracting the values of a blank sample. These results are given in arbitrary units because the intensity of fluorescence is acquired with an automatic optimal gain and hence varies from one experiment to the other.

### Lipid extraction and quinone analysis

Cultures of 2, 5, or 10 mL were cooled on ice for at least 30 minutes before centrifugation at 3,200× *g* at 4°C for 10 minutes. Cell pellets were washed in 1-mL ice-cold PBS and transferred to pre-weighted 1.5-mL Eppendorf tubes. After centrifugation at 12,000× *g* at 4°C for 1 minute, the supernatant was discarded, the cell wet weight was determined, and pellets were stored at −20°C until lipid extraction, if necessary. Quinone extraction from cell pellets was performed as previously described (6). The dried lipid extracts were resuspended in 100 µL ethanol, and a volume corresponding to 1 mg of cell wet weight was analyzed by high performance liquid chromatography (HPLC) electrochemical detection-mass spectrometry (ECD-MS) with a BetaBasic-18 column at a flow rate of 1 mL/minute with a mobile phase composed of 50% methanol, 40% ethanol, and 10% of a mix (90% isopropanol, 10% ammonium acetate [1 M], and 0.1% formic acid). When necessary, MS detection was performed on an MSQ spectrometer (Thermo Scientific) with electrospray ionization in positive mode (probe temperature, 400°C; cone voltage, 80 V). Single-ion monitoring detected the following compounds: UQ_8_ (*M*+H^+^), *m*/z 727–728, 6–10 minutes, scan time of 0.2 seconds; 3(^18^O)-UQ_8_ (*M*+H^+^), *m*/z 733–734, 6–10 minutes, scan time of 0.2 seconds; UQ_8_ (*M*+NH_4_^+^), *m*/z 744–745, 6–10 minutes, scan time of 0.2 seconds; and UQ_10_ (*M*+NH_4_^+^), *m*/z 880–881, 10–17 minutes. MS spectra were recorded between *m*/z 600 and 900 with a scan time of 0.3 seconds. ECD and MS peak areas were corrected for sample loss during extraction on the basis of the recovery of the UQ_10_ internal standard and then were normalized to cell wet weight. The peaks of UQ_8_ obtained with electrochemical detection or MS detection were quantified with a standard curve of UQ_10_ as previously described (6).

### ^18^O_2_ labeling

MG1655 wild type (wt) and Δ*ubiI*Δ*ubiH*Δ*ubiF* containing, respectively, the pBAD24 empty vector or pBAD-*ubiUV* were grown overnight at 37°C in LB medium supplemented with ampicillin (100 µg/mL) and 0.05% arabinose. These precultures were used to inoculate 20 mL of the same fresh medium at an OD_600_ of 0.05 in Erlenmeyer flasks of 250 mL. The cultures were grown at 37°C, 180 rpm, until an OD_600_ of 0.4–0.5 was reached. An aliquot was taken for lipid extraction and quinone analysis (0 minute of ^18^O_2_), and 13 mL of each culture was transferred to an Hungate tube. Five milliliter of labeled molecular oxygen (^18^O_2_) was injected through the septum with disposable syringes and needles, and the incubation was continued at 37°C, 180 rpm for 2 hours. Then 5 mL of each sample was taken for quinone analysis (120 minutes of ^18^O_2_).

### Mouse intestine colonization experiments

Four-week-old female BALB/cByJ were purchased from Charles River Laboratories (Saint-Germain-Nuelles) and were acclimatized in a controlled animal facility under specific pathogen-free conditions for 2 weeks prior to the beginning of the colonization assay. Mice were randomly assigned to groups of three or five per cage, and ear punching was used to identify each mouse in a given cage.

The colonization experiments were adapted and performed as previously described (25, 26). Mice were given drinking water containing streptomycin sulfate and glucose (both 5 g/L) for 72 hours to remove existing resident anaerobic facultative microflora. For the clearance of streptomycin, freshwater devoid of antibiotics and glucose was then given to mice for 48 hours before the inoculation of *E. coli* strains and for the rest of the experiment. To start the competition experiment, the mice were orally inoculated with 200 µL of a mixture in a 1:1 ratio of the two competing strains at ~20,000 cells/mL in PBS. Mice from each cage were orally inoculated with the same solution of bacteria. An aliquot of inoculum was plated on LB agar containing 15 µg/mL tetracycline to compute the input value.

The relative abundance of both competing strains was then monitored for several days post-inoculation in fecal samples. Fecal samples were collected from each mouse in pre-weighed 1.5-mL Eppendorf tubes containing the equivalent of 100-µL glass beads (diameter 0.25–0.5 mm) and 80-µL PBS, and the feces weight was determined. A volume of PBS was then added to each tubeto obtain a final concentration of 0.15 g of feces per 1-mL PBS. The feces were homogenized by vortexing for 2 minutes, serially diluted by 10-fold steps up to a 10^5^-fold dilution, and aliquots of 70 µL were plated on LB agar medium containing 15 µg/mL tetracycline. The plates were incubated overnight at 37°C and were transferred at 4°C for at least 2 hours the following day, before imaging under blue light which revealed the fluorescent markers carried by each colony. The red and green colonies corresponding, respectively, to MP7 and MP13 strains were counted by an adapted version of ImageJ. Then, the CFU was computed per gram of feces for each strain and a competitive index (CI) was calculated as a ratio of (MP13 mutant CFU/MP7 wt CFU)/(input MP13 mutant CFU/input MP7 wt CFU), where the input CFU was determined from the inoculum for which an aliquot was plated on the day of gavage. The limit of detection in fecal plate counts was 10^2^ CFU/g feces. At all time points, the wt strain was detectable on the fecal plates. The absence of CFU count and CI for 1 day in one mouse corresponds to the absence of feces for that day. Significance of CI was calculated by GraphPad Prism using one sample *t*-test compared to one.

## RESULTS

### Biochemical function of UbiUV *in vivo*

**Fig 1:**
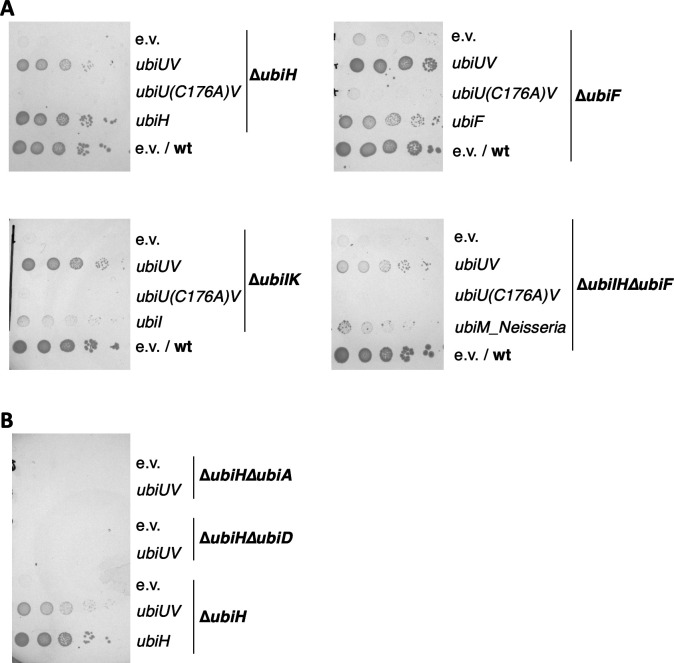
Complementation of *ubiI*, *ubiH*, and *ubiF* mutants by pBAD-*ubiUV* in the presence of O_2_. (**A**) *E. coli* mutant strains ∆*ubiH* (FBE253), ∆*ubiF* (FBE512), ∆*ubiIK* (FBE713), and ∆*ubiIH∆ubiF* (FBE650) were transformed by pBAD24 (e.v.), pBAD-UbiUV, and pBAD-UbiU(C176A)V plasmids. (**B**) *E. coli* mutant strains ∆*ubiH∆ubiA* (FBE792), ∆*ubiH∆ubiD* (FBE793), and ∆*ubiH* (FBE253) were transformed by pBAD24 (e.v.) and pBAD-UbiUV. (**A and B**) After selection in the absence of O_2_, cultures were washed and serially diluted in minimal medium and then spotted on M9 succinate plates containing 0.02% arabinose and incubated at 37°C for 48 hours (or 96 hours for the ∆*ubiIHF* series) in aerobic conditions (21% O_2_). The results shown are representative of at least two independent experiments.

To get further insight into the UbiUVT system *in vivo*, we tested whether the overproduction of UbiU and UbiV could substitute for the three oxygen-dependent hydroxylases UbiI, UbiH, or UbiF. Thus, we cloned the *ubiUV* operon in the pBAD24 vector downstream the arabinose-inducible pBAD promoter (pES154 plasmid). In parallel, we also cloned *ubiUV* upstream the SPA tag encoding sequence to assess the quantities of proteins produced. The pBAD-*ubiUV*-SPA plasmid produces a level of UbiV protein approximately 30-fold higher than that produced by a chromosomal copy of *ubiV-SPA* under anaerobiosis (Fig. S2). After the transformation of mutant strains, selection, and precultures with LB medium in absence of O_2_, growth on M9 succinate was tested as it strictly depends on an aerobic UQ-dependent respiratory chain (Fig. 1). In the presence of an inducer, the pES154 plasmid was able to suppress the growth phenotype of the ∆*ubiF*, ∆*ubiH*, ∆*ubiIK*, and ∆*ubiIHF* mutants (Fig. 1A). Note that as a control, we used the *Neisseria meningitidis ubiM* gene that we previously showed to substitute for the growth phenotype of a ∆*ubiIHF* mutant (19). Also, in M9 succinate, the ∆*ubiI* mutation alone has no growth phenotype and needs to be combined with ∆*ubiK* mutation for a defect to be observed (27). To test the importance of the UbiU-bound [Fe–S] cluster, a complementation test was carried out in the same conditions, using a pBAD derivative carrying the *ubiU*(C176A) allele that produces an UbiU variant lacking its [Fe–S] cluster (3). Accordingly, the suppression of ∆*ubiH*, ∆*ubiF*, ∆*ubiIK*, and ∆*ubiIHF* was no longer observed (Fig. 1A). In addition, the pES154 plasmid was unable to suppress the growth phenotype of ∆*ubiA*, ∆*ubiD*, ∆*ubiE*, or ∆*ubiG* strains (data not shown) and was also unable to suppress the growth phenotype of ∆*ubiH*∆*ubiA* or ∆*ubiH*∆*ubiD* mutants (Fig. 1B), showing that UbiUV intervene specifically at the hydroxylation steps and otherwise depend on all the other components of the aerobic UQ biosynthesis pathway to do so. These results indicate that in the presence of O_2_, expression of UbiUV can substitute for the O_2_-dependent UbiIHF hydroxylases and that integrity of the UbiU [Fe–S] cluster is required.

**Fig 2:**
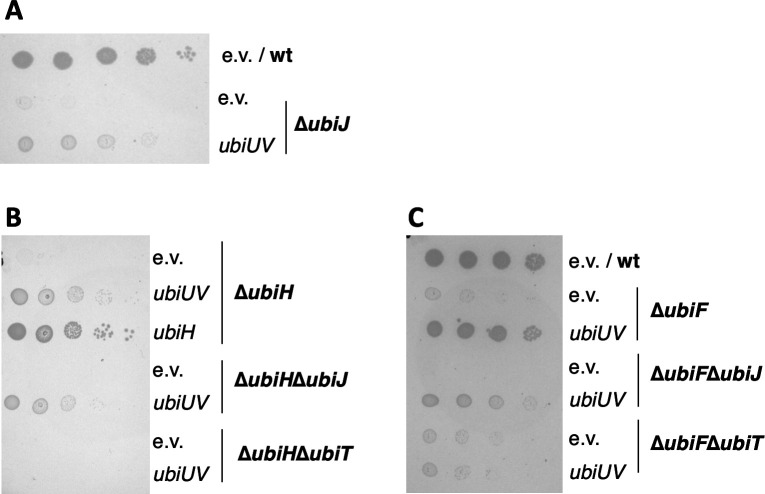
Complementation of *ubiH* and *ubiF* mutants by pBAD-UbiUV is *ubiT* dependent (and *ubiJ* independent). *E. coli* mutant strains were transformed by pBAD24 (e.v.) and pBAD-UbiUV plasmids. After selection in the absence of O_2_, cultures were washed and serially diluted in minimal medium and then spotted on M9 succinate plates containing 0.02% arabinose at 37°C in aerobic conditions. The results shown are representative of at least two independent experiments. (**A**) Strains ∆*ubiJ* (FBE514) and wt; (**B**) strains ∆*ubiH* (FBE253), ∆*ubiH*∆*ubiJ* (FBE794), and ∆*ubiH*∆*ubiT* (FBE795); (**C**) strains ∆*ubiF* (FBE512), ∆*ubiF*∆*ubiJ* (FBE264), ∆*ubiF*∆*ubiT* (FBE265), and wt.

Remarkably, the expression of the pES154 plasmid was also able to suppress growth defects of the ∆*ubiJ* mutant (Fig. 2A). UbiJ is an auxiliary factor important for organizing the aerobic Ubi metabolon. We reasoned that suppression was made possible thanks to the presence of the chromosomally encoded UbiT that shares sequence similarity with UbiJ. To test this, we repeated the complementation test in two new strains, ∆*ubiH*∆*ubiJ* and ∆*ubiH*∆*ubiT*. The pES154 plasmid still complemented the growth defects of the ∆*ubiH*∆*ubiJ* mutant, but it was unable to complement the ∆*ubiH*∆*ubiT* mutant (Fig. 2B). Similarly, pES154 was found to suppress the growth defect phenotype of a ∆*ubiF*∆*ubiJ* mutant but not a ∆*ubiF*∆*ubiT* mutant (Fig. 2C). These results showed that in the presence of O_2_, increased dosage of *ubiUV* genes suppresses the lack of O_2_-dependent hydroxylases UbiF and UbiH in an UbiT-dependent/UbiJ-independent manner.

**Fig 3:**
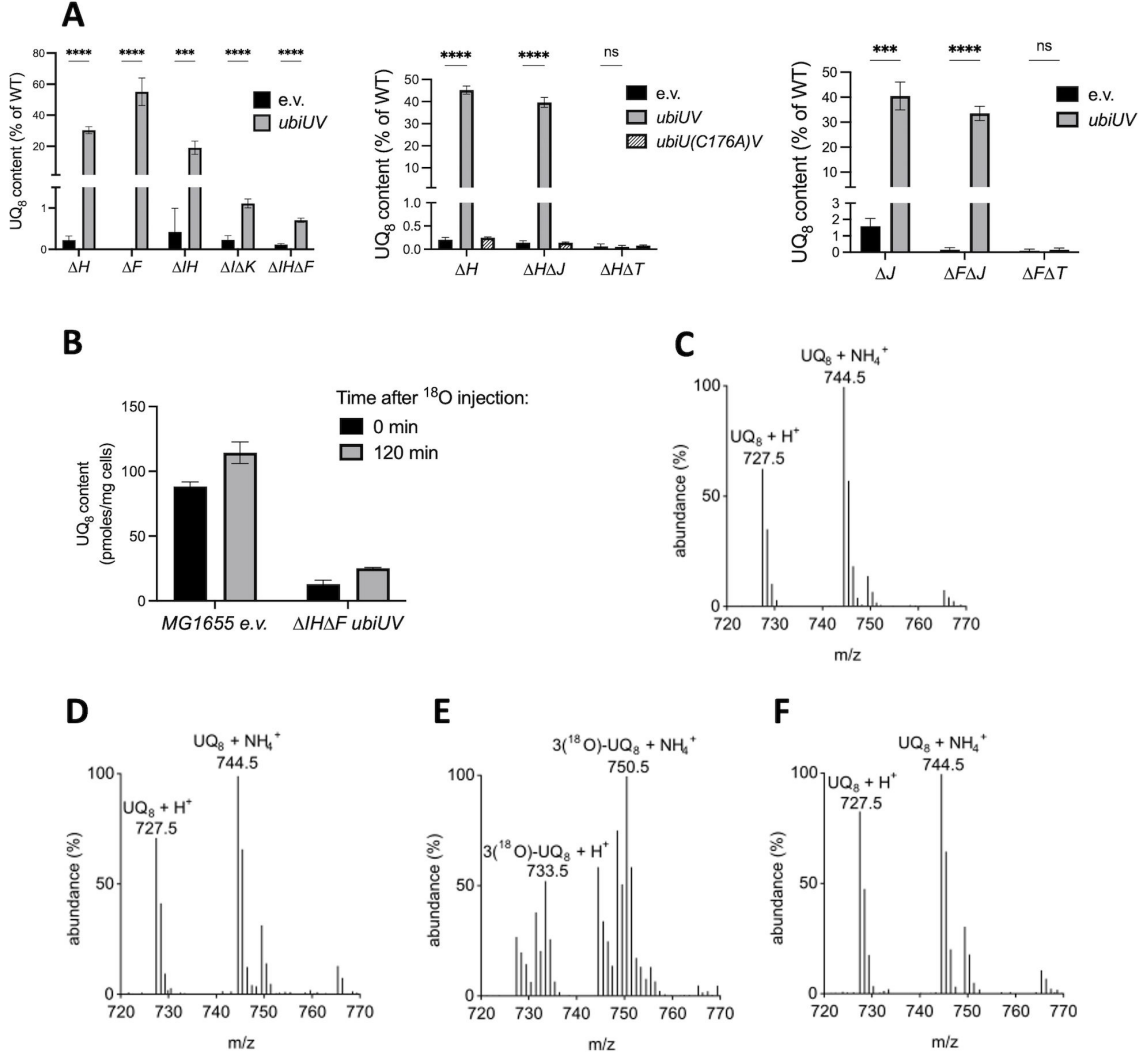
UbiUV restores the UQ_8_ content of ∆*ubiIH* and ∆*ubiF* mutants without using O_2_ for the hydroxylation steps. (**A**) UQ_8_ content of the indicated strains containing either pBAD (e.v.), pBAD-*ubiUV*, or pBAD-*ubiU(C176A)V* after aerobic growth overnight at 37°C in LB medium. Mean ± SD (*n* = 3 to 4). ***P < 0.001; ****P < 0.0001 by unpaired Student’s *t*-test. (**B–F**) Detection of UQ_8_ with ^18^O_2_ labeling. (**B**) Quantification of UQ_8_ content in wt (MG1655) cells containing an empty vector (e.v.) and in Δ*ubiIH*∆*ubiF cells* containing the pBAD-*ubiUV* vector just before (0 minute) and 2 hours (120 minutes) after adding ^18^O_2_. Mean ± SD (*n* = 2). (C–F) Mass spectra of UQ_8_ from cells shown in B, wt (**C and E**), and Δ*ubiIH*∆*ubiF* with pBAD-*ubiUV* (**D and F**), before (**C and D**) and 2 hours after (**E and F**) the addition of ^18^O_2_. Mass spectra representative of two independent experiments.

To confirm that phenotypic suppression was due to UQ_8_ synthesis, we quantified the UQ_8_ content by HPLC analysis coupled to electrochemical detection (ECD) for all strains described above (Fig. 3A). Results showed that mutant strains lacking UbiI-UbiK, UbiH, and/or UbiF were severely deficient in UQ. The pES154 plasmid-enabled ∆*ubiH*, ∆*ubiF*, or ∆*ubiIH* strains to synthesize 30%–50% of the UQ level of the wt strain (Fig. 3A, first panel). The levels of UQ obtained in the ∆*ubiIH*∆*ubiF* and ∆*ubiIK* mutant strains with the pES154 plasmid were much lower. We stress that the UQ levels cannot be directly correlated with the phenotypic analysis (Fig. 1 and 2) since culture media were different (LB versus M9 succinate) to allow the recovery of enough biological material for the HPLC-ECD analyses. Importantly, the pBAD-*ubiU(C176A)V* plasmid was unable to promote UQ synthesis in ∆*ubiH* (Fig. 3A, second panel). Last, UQ_8_ content assay confirmed that UbiT, but not UbiJ, was necessary for UbiUV to synthesize UQ in aerobic conditions (Fig. 3A, third panel).

The results above showed that UbiUV hydroxylate UQ precursors, when expressed under aerobic conditions. This result raised the possibility that under such conditions, O_2_ might be used as a co-substrate of the hydroxylation reactions, as is the case for UbiI, UbiH, and UbiF in wt cells (5). To test this hypothesis, we exposed cells to ^18^O_2_ and monitored the labeling of UQ by HPLC-ECD-MS. Two hours after ^18^O_2_ addition, the level of UQ_8_ increased in both strains (Fig. 3B). Before adding ^18^O_2_, the mass spectra of UQ synthesized by wt or Δ*ubiIH*Δ*ubiF* cells containing pES154 displayed H^+^ and NH_4_^+^ adducts with *m*/z ratio characteristic of unlabeled UQ (Fig. 3C and D). As expected, 2 hours after adding ^18^O_2_, most of the UQ_8_ pool in wt cells contained three ^18^O_2_ atoms (Fig. 3E), in agreement with O_2_ being the co-substrate of the aerobic hydroxylation steps (5). In contrast, we detected only unlabeled UQ_8_ in the Δ*ubiIH*Δ*ubiF* strain expressing UbiUV (Fig. 3F), demonstrating that UbiUV utilizes another oxygen donor than O_2_, even when operating under aerobic conditions.

Altogether, both phenotypic and UQ_8_ quantification results allowed us to conclude that UbiU and UbiV, when produced at sufficiently high level, function in the canonical “aerobic” UQ_8_ biosynthesis pathway by catalyzing [Fe–S]-dependent hydroxylation of the benzene ring in an O_2_-independent reaction. Remarkably, UbiT is necessary for such aerobic UbiUV-mediated synthesis to occur and cannot be substituted by UbiJ.

### The ISC [Fe–S] biogenesis machinery is required for anaerobic UQ biosynthesis

The UbiU and UbiV proteins each contain a [4Fe–4S] cluster, which is essential for the synthesis of UQ_8_ in anaerobic conditions (3). Assembly of [4Fe–4S] clusters requires complex biosynthetic machineries, ISC and SUF (28). Therefore, the UQ_8_ levels were monitored in Δ*isc* and Δ*suf* mutants grown in anaerobic conditions (Fig. S3). UQ_8_ content in Δ*isc* mutants was strongly impaired (around 15% of the wt), while it was much less affected in ∆*suf* mutants (60%–80% of the wt). This indicated that the ISC system contributes to anaerobic UQ_8_ biosynthesis likely through the maturation of [4Fe–4S] clusters in UbiU and UbiV. An alternative explanation would be that isopentenyl phosphate (IPP), which is the precursor of UQ_8_ and whose synthesis depends on [4Fe–4S] containing IspG and IspH proteins, is not efficiently synthesized in the Δ*isc* mutants. However, DMK_8_ and MK_8_ levels, which also rely on IspG/IspH-synthesized IPP, remained mostly unaltered in the Δ*isc* mutants. It is likely that in this case, the SUF system takes over in a more efficient way as it does for maturating UbiU and UbiV proteins. Collectively, these results showed that the ISC system and to some minor extent the SUF system are necessary for anaerobic UQ_8_ biosynthesis.

### Anaerobic and microaerobic UQ biosynthesis

**Fig 4:**
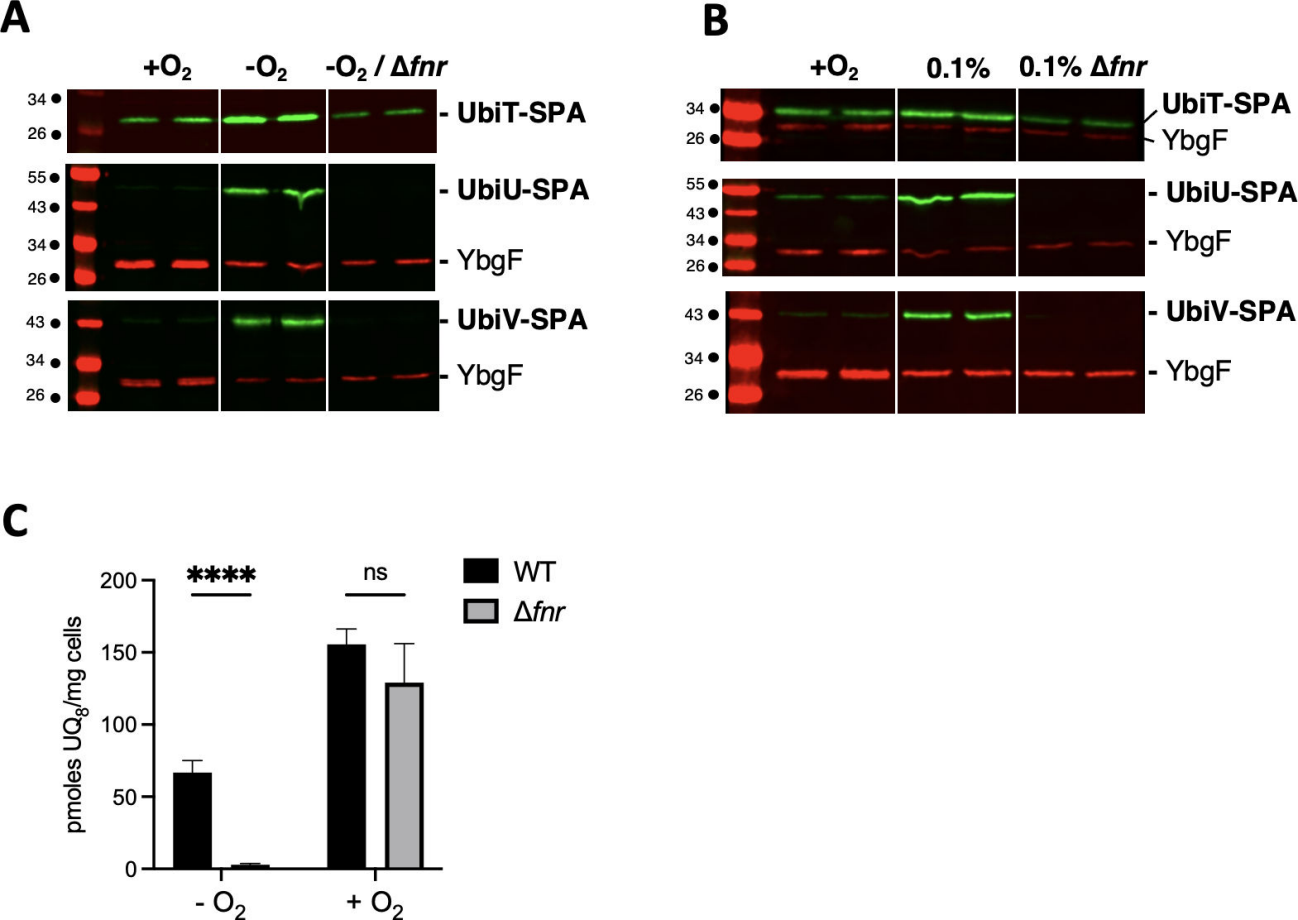
Fnr controls UbiTUV expression and UQ biosynthesis under anaerobiosis. (**A and B**) *E. coli* strains UbiU-SPA, UbiV-SPA, and UbiT-SPA, and their corresponding ∆*fnr* versions (strains FBE656, FBE789, FBE655, FBE695, FBE696, and FBE694) were grown in LB at 37°C in +O_2_ and –O_2_ (**A**) or in +O_2_ and 0.1% O_2_ (**B**) and analyzed by western blot: normalized quantities of total protein extracts (in biological duplicate) were separated by SDS-PAGE 12% and detected by western blot using anti-Flag monoclonal antibody for the detection of the SPA tag (green) or anti-YbgF polyclonal antibodies as an internal loading control (red). (**C**) UQ_8_ content of the wild-type and ∆*fnr* (FBE354) strains was assayed after aerobic or anaerobic growth overnight at 37°C in LB medium. Mean ± SD (*n* = 3–4). ****P < 0.0001 by unpaired Student’s *t*-test.

Genome-scale studies have predicted that *ubiUV* genes are under the control of the anaerobic Fnr transcriptional activator (29, 30). In contrast, *ubiT* did not appear as a potential Fnr target. This prompted us to investigate the effect of anaerobiosis (0% O_2_), microaerobiosis (0.1% O_2_), and aerobiosis (21% O_2_) on the level of UbiU, UbiV, and UbiT proteins. To follow the quantity of UbiTUV proteins in physiological conditions, we constructed a series of recombinant strains producing the UbiT, UbiU, or UbiV proteins with a C-terminal SPA tag (14) encoded from a gene fusion at their chromosomal loci. We examined protein production by western blot assay using an anti-flag antibody and assessed loading with a polyclonal antibody against YbgF (CpoB). All three UbiTUV-SPA tagged proteins were present in strains grown in anaerobiosis (Fig. 4A) and microaerobiosis (Fig. 4B). In aerobiosis, the production of UbiU and UbiV was no longer observed, whereas a significant level of UbiT was still visible. The contribution of Fnr to anaerobiosis- or microaerobiosis-mediated activation of *ubiU* and *ubiV* genes was confirmed as no cognate UbiU or UbiV-associated band was observed in a ∆*fnr* mutant (Fig. 4A and B). Interestingly, UbiT level was also reduced in the ∆*fnr* mutant in −O_2_. Last, to validate the physiological significance of the Fnr regulatory circuit depicted above, we quantified the amount of UQ_8_ produced in wt and ∆*fnr* strains, during aerobiosis and anaerobiosis (Fig. 4C). In comparison to the UQ content found in the wt strain in aerobiosis, the level in anaerobiosis was reduced by half. Importantly, we observed that almost no UQ was detected in the ∆*fnr* mutant (Fig. 4C). This revealed the pivotal role that Fnr plays in allowing UQ_8_ synthesis in the absence of O_2_.

### Genetic control of *ubiUVT* gene expression

**Fig 5:**
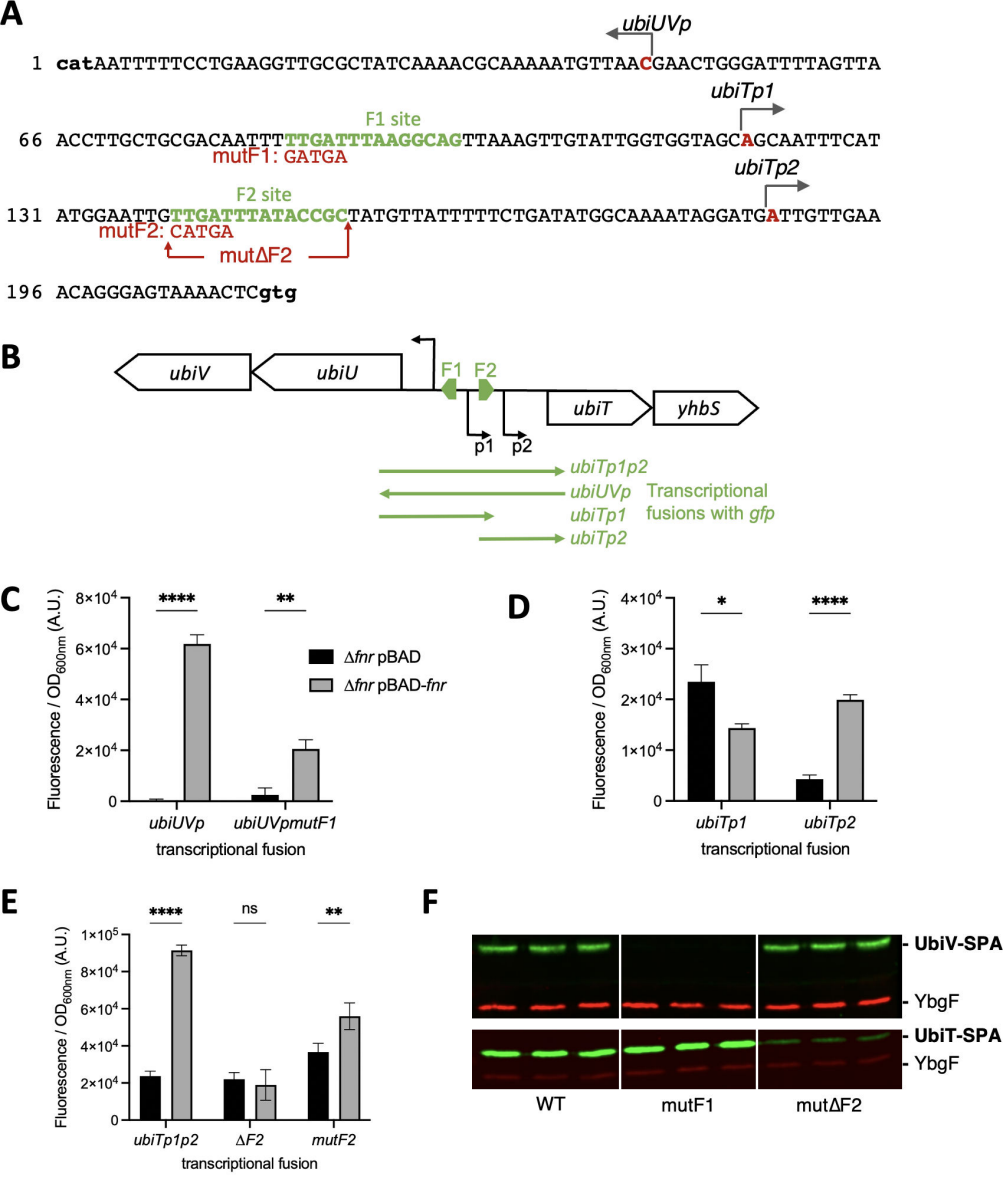
(**A**)Organization of the promoter region of *ubiTUV* genes. The sequence of the intergenic region between *ubiU* and *ubiT* genes is shown, from the start codon of *ubiU* to the start codon of *ubiT* (both indicated in bold at the extremities of the sequence). The transcription start sites as determined in (31) are indicated in red. The two Fnr-binding sites F1 and F2 are indicated in green. The mutF1, mutF2, and mut∆F2 mutations introduced in the transcriptional fusions are depicted in red. (**B**) Limits of transcriptional fusions used in panels C–E. (**C–E**) Activity of the transcriptional fusions with or without *fnr* expression. ∆*fnr E. coli* strain was co-transformed by pBAD24 or pBAD-*fnr* together with the indicated transcriptional fusions. After overnight growth of four biological replicates at 37°C in LB in anaerobiosis, GFP levels were quantified. Errors bars indicate the SD. *P < 0.1; **P < 0.01; ****P < 0.0001 by unpaired Student’s *t*-test. (**F**)Role of the Fnr sites in UbiTUV physiological levels. *E. coli* strains UbiV-SPA and UbiT-SPA, without (wt) or with the indicated mutation in the F1- or F2-binding sites, were grown in LB overnight at 37°C in the absence of O_2_. Normalized quantities of total protein extracts (in biological triplicate) were separated by 12% SDS-PAGE and detected by western blot using anti-Flag monoclonal antibody for the detection of the SPA tag (green) or anti-YbgF polyclonal antibody as an internal loading control (red).

Previous genome Chip-seq analysis reported binding of Fnr within the *ubiT–ubiUV* intergenic region. Additionally, in a whole-genome sequence search study, one transcription start site has been described upstream of the *ubiUV* operon (*ubiUV_p_*) and two sites described upstream of *ubiT* (*ubiT_p_*_1_, *ubiT_p_*_2_) (31) (Fig. 5A and B). On inspection of that region, we were able to identify two potential Fnr-binding sites fitting well with the described Fnr-binding consensus. The F1 site, reading TTGATTTAAGGCAG is located 36 nucleotides (nt) upstream the *ubiUV_p_* transcription start site (Fig. 5A). The F2 site reading TTGATTTATACCGC locates 33 nt upstream the proximal +1 transcription starting site *ubiT_p2_* and 19 nt downstream the distal *ubiT_p1_* (Fig. 5A and B).

To detail the molecular mechanism of regulation and to dissect the promoter organization of the intergenic region between *ubiUV* and *ubiT*, we used transcriptional fusions with GFP (23). We used four different transcriptional fusions encompassing *ubiUV_p_*, *ubiT_p_*_1_, *ubiT_p_*_2_, and a construction *ubiT_p_*_1_*_p_*_2_ containing the two promoters of *ubiT* (Fig. 5B). We compared the expression of these transcriptional fusions in anaerobiosis, in a ∆*fnr* mutant complemented or not with a pBAD-*fnr* plasmid. The *ubiUV_p_* and *ubiT_p_*_2_ promoters were strongly activated in the presence of pBAD-*fnr*, whereas the *ubiT_p_*_1_ promoter was not (Fig. 5C and D). This suggested that the *ubiUV_p_* promoter was activated by Fnr binding to the F1 site and that the *ubiT_p_*_2_ promoter was activated by Fnr binding to the F2 site. When we introduced mutations in the F1-binding site (five mutated nucleotides; mutF1; Fig. 5A), the activation of the expression from the *ubiUVp* transcriptional fusion was severely reduced (Fig. 5C). Mutations of the F2 site (mut∆F2 complete deletion or mutF2 with five mutated nucleotides; Fig. 5A) also affected the expression of the ubiT*_p_*_1_*_p_*_2_ transcriptional fusion, but a basal level of expression was maintained, probably due to the expression from the distal *ubiT_p_*_1_ promoter (Fig. 5E).

Next, we introduced the same mutations in the F1 and F2 Fnr-binding sites at the locus in the *ubiU–ubiT* intergenic region in the chromosome of the strains producing UbiV-SPA or UbiT-SPA tagged proteins. Mutation within the F1 site upstream *ubiU* completely prevented the production of UbiV in the absence of O_2_ (Fig. 5F). Mutation within the F2 site upstream the proximal *ubiT_p_*_2_ promoter prevented the induction of *ubiT* in the absence of O_2_, without altering the basal level of UbiT-SPA observed in the presence of O_2_ (Fig. 5F; Fig. S4). Notably, the mutation in the F1-binding site did not affect the expression of *ubiT* and conversely, the mutation of the F2-binding site did not affect the expression of *ubiV*.

Altogether, these results showed that Fnr activates *ubiUV* transcription under anaerobiosis, while *ubiT* expression can be triggered from two promoters, one aerobically active (P1) and the other anaerobically active (P2) under Fnr control.

### Physiological role of UbiUVT at different O_2_ levels

We have previously reported that UbiU, UbiV, and UbiT are essential for the anaerobic synthesis of UQ in *E. coli* when grown in LB, glycerol/DMSO, or lactate/NO_3_^−^ (3). However, the contribution to *E. coli* physiology of UQ synthesized by UbiUVT in anaerobic conditions was not investigated in detail. We made use of a set of mutants altered in aerobic (*ubiH*) or anaerobic (*ubiUV*, *ubiT*) UQ_8_ synthesis, as well as mutants altered in DMK/MK biosynthesis (*menA*) to assess the contribution of each type of quinone for growth in a wide range of O_2_ level, 21% (aerobic), 0.1% (microaerobic), and 0% O_2_ (anaerobic), and with varying carbon sources (e.g., glycerol or glucose) and electron terminal acceptors (e.g., O_2_ or NO_3_^−^).

**Fig 6:**
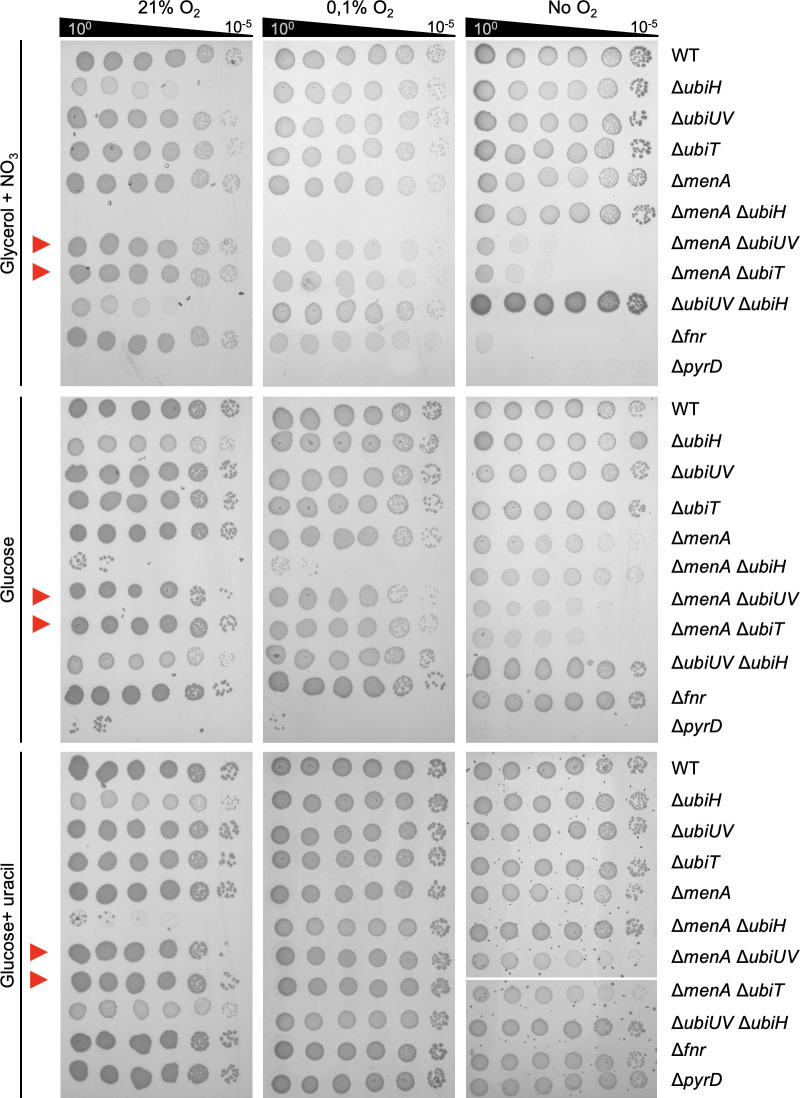
Role of *ubiUVT* in anaerobic growth. *E. coli* wt and strains devoid of the MK/DMK *(*∆*menA)*, UQ synthesis pathways—aerobic (∆*ubiH*) and anaerobic (∆*ubiUV* or ∆*ubiT*)—and controls for anaerobic growth (∆*fnr*) and auxotrophy for uracil (∆*pyrD*) were grown aerobically at 37°C in LB medium or LB glucose 0.2% (for ∆*menA* ∆*ubiH*), washed, and resupended in M9 medium without carbon source to OD_600_ of 1. Serial dilutions were spotted in agarose M9 medium plates supplemented with carbon source (glycerol or glucose), KNO_3_, or uracil and incubated at 37°C at the indicated O_2_ concentration until growth was observed. Experiments were performed in triplicates and confirmed with at least four independent biological replicates.

In the presence of glycerol and NO_3_^−^ under aerobic conditions (Fig. 6, upper left panel), Δ*ubiUV* and Δ*ubiT* strains showed no growth phenotype. In such conditions, while NO_3_ is present, O_2_ is used for respiration. This contrasted with the Δ*ubiH* mutant, which was severely affected. Combining Δ*ubiH* and Δ*ubiUV* bore no aggravating effect. In contrast, combining both ∆*ubiH* and ∆*menA* had an aggravating effect, indicating that in addition to UQ, DMK and/or MK can support *E. coli* growth even in aerobiosis, as previously suggested (32). In microaerobic conditions (Fig. 6, upper center panel), no phenotype was observed for Δ*ubiUV* or Δ*ubiT* strains. In contrast, the Δ*menA* Δ*ubiH* strain still exhibited a clear defect, suggesting that *ubiUV* and *ubiT* do not bear a prominent role in NO_3_^−^-dependent respiratory metabolism under microaerobic conditions, despite being expressed in microaerobiosis (see above). This notion was also supported by the fact that at 0.1% O_2_, the Δ*ubiH* and Δ*ubiH*Δ*ubiUV* strains did not show any phenotype. At 0.1% O_2_, UQ-dependent metabolism through cytochrome *bd* or *bo* oxidases would remain inconsequential, and cells presumably rely on DMK/MK-dependent metabolism for anaerobic respiration (33). Last, in anaerobic conditions, with NO_3_^−^ used for respiration, Δ*ubiUV*, Δ*ubiT*, and Δ*menA* strains showed wt-like growth phenotype (Fig. 6, upper right panel). However, combining Δ*menA* and Δ*ubiUV* mutations or Δ*menA* and Δ*ubiT* mutations drastically hampered NO_3_^−^ respiratory capacities. In fact, the growth of these mutants on M9 glycerol NO_3_^−^ was barely better than a Δ*fnr* strain (Fig. 6), which was used as a control since it was shown that such strain is unable to respire nitrate but can still use glucose anaerobically (34). These results indicated that anaerobically UbiUVT-synthesized UQ and MK are fully interchangeable electron carriers during NO_3_^−^ respiration under full anaerobiosis (35). Furthermore, we could exclude that the aerobic UQ biosynthetic pathway could contribute to growth in such conditions as the Δ*ubiH* and Δ*menA* Δ*ubiH* mutants exhibited no growth phenotype.

In the presence of glucose as a carbon source and under aerobiosis, Δ*ubiUV* and Δ*ubiT* mutants exhibited wt-like growth capacity (Fig. 6, left middle panel). The ∆*ubiH* mutant showed some slower growth, but a most spectacular negative additive effect was observed on combining ∆*ubiH* and Δ*menA* mutations. It likely points out a role for DMK/MK in aerobic electron transport (36). In anaerobiosis, neither ∆*ubiH* nor ∆*menA*, alone or in combination, showed defect in the presence of glucose as a carbon source (Fig. 6, middle right panel). In contrast, ∆*menA* ∆*ubiUV* or ∆*menA* ∆*ubiT* mutants exhibited additive growth defects (Fig. 6, middle right panel). This indicated that the UbiUVT-biosynthesized UQ was crucial for growth in glucose fermentative conditions, in the absence of MK. A possibility was that this negative effect reflected auxotrophy for uracil, whose synthesis depends on electron transfer from PyrD dihydrooratate dehydrogenase to fumarate reductase (FrdABCD) via quinones in anaerobiosis (37). As a matter of fact, adding uracil to the medium had a rescuing effect (Fig. 6, lower right panel), supporting the notion that uracil deficiency was responsible for the growth defect observed in the ∆*ubiUV* ∆*menA* mutant in anaerobiosis. This was an important observation as early studies had proposed that the PyrD/FrdABCD electron transfer chain relied mostly on MK/DMK and marginally, if at all, on UQ (37). Our observation clearly shows that anaerobically synthesized UQ can also allow the functioning of PyrD. Incidentally, we noticed that the addition of uracil did not rescue the growth defect of the ∆*menA* ∆*ubiH* mutant in aerobiosis, but we have no explanation for this observation.

### Contribution of the O_2_-independent UQ biosynthesis pathway to mouse intestine colonization

**Fig 7:**
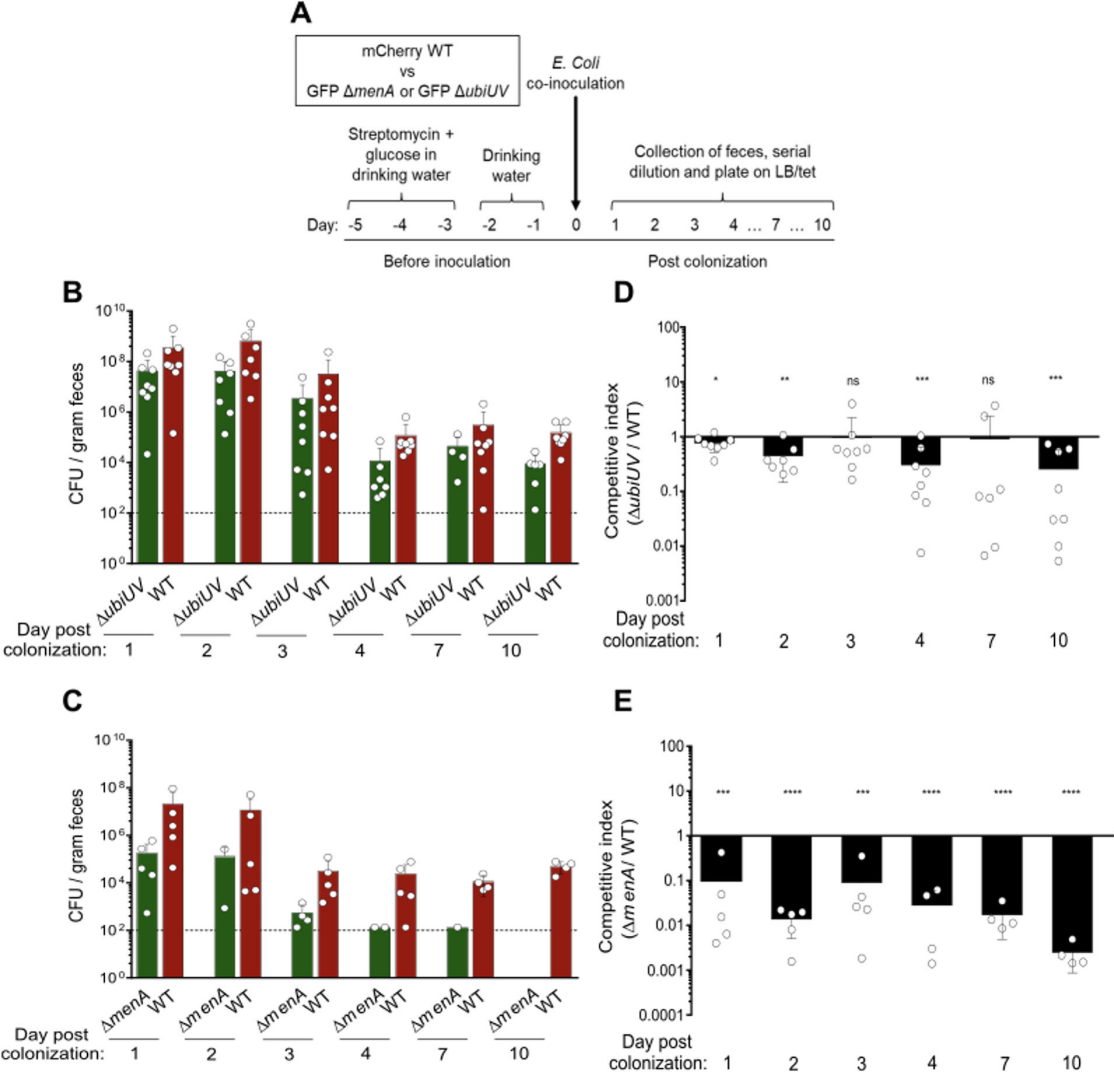
Quinones contribute differently to the colonization of the mouse gut by *E. coli*. (**A**) Schematic representation of the experimental protocol for the mouse intestine colonization competitions, adapted from Ref. (25). (**B and C**) Total CFU counts (**B**) and associated competitive index (CI) (**C**) in feces of mice after oral co-inoculation with a 1:1 ratio of MP7 wt and MP13 Δ*ubiUV* strains. (**D and E**) Same as panels B and C with MP7 wt and MP13 Δ*menA* strains. The limit of detection of 10^2^ CFU is indicated as dotted line. Mean ± SD, each white circle represents values for individual mice (*n* = 5 and 8), circles missing corresponds to the absence of feces for that day. ns, not significant; *P < 0.05; **P < 0.01; ***P < 0.001; ****P < 0.0001 by one sample *t* test. Changes in total CFU counts and CI throughout the experiment in each mouse are shown in Fig. S6.

Since enterobacteria evolve mostly in anaerobic conditions in their natural habitat, we evaluated the physiological importance of the O_2_-independent UQ biosynthesis pathway in the mouse intestine. To do so, we performed competition experiments between two isogenic strains, MP7 and MP13, which respectively express mCherry and GFP in the presence of tetracycline (22). We deleted *ubiUV* in the MP13 background and confirmed, as expected, that this strain was deficient for UQ_8_ when grown anaerobically (Fig. S5A). MK was previously shown to be important for the efficient colonization of the mouse intestine by *E. coli* (38). Thus, we also constructed a ∆*menA* mutant in the MP13 background. We checked that the deletion of ∆*menA* abrogated the synthesis of DMK and MK (Fig. S5B and C). The fitness of the ∆*ubiUV* and ∆*menA* mutants was tested in competition experiments with the MP7 wt strain. We monitored the abundance of each strain in the feces of mice up to 10 days after co-inoculation by oral gavage (Fig. 7A). In both experiments, the total CFU count reached ~10^8^/g of feces 24-hour post-inoculation (Fig. 7B and C; Fig. S6A and B) and then gradually decreased to ~10^5^, showing efficient colonization of the MP7 strain. The abundance of the *ubiUV* mutant was slightly decreased compared to wt (Fig. 7B; Fig. S6A), which translated into an average CI <1 (Fig. 7D; Fig. S6C) at days 1, 2, 4, and 10. We noticed, however, a rather high inter-individual variability (Fig. S6C). In contrast, the ∆*menA* mutant was markedly less abundant than the wt (Fig. 7C; Fig. S6B) and was even undetectable at day 10. CI <1 were observed for every mouse at every sampling (Fig. 7E; Fig. S6D), and the values obtained were much lower than in the case of the ∆*ubiUV* mutant. Collectively, these data confirm that DMK/MK is the most important quinone for the physiology of *E. coli* in the mouse intestine (38). However, they also reveal a contribution, albeit minor, of the O_2_-independent UbiUV-mediated UQ biosynthesis pathway.

### Role of UbiT within the anaerobiosis–aerobiosis shift

Phenotypic analysis above revealed that anaerobically UbiUVT-synthesized UQ_8_ was contributing to growth via glucose fermentation or NO_3_^−^ respiration. In both conditions, anaerobic UbiUVT-synthesized UQ_8_ was functionally redundant with anaerobically synthesized DMK/MK. Because UQ_8_ is crucial under aerobiosis, we wondered whether anaerobically synthesized UQ_8_ might prepare the cells to adapt to an aerobic environment, that is, before the aerobic UbiIHF-dependent synthesis takes over. Thus, we investigated the role of UbiUVT-synthesized UQ_8_ in the anaerobiosis–aerobiosis transition.

**Fig 8:**
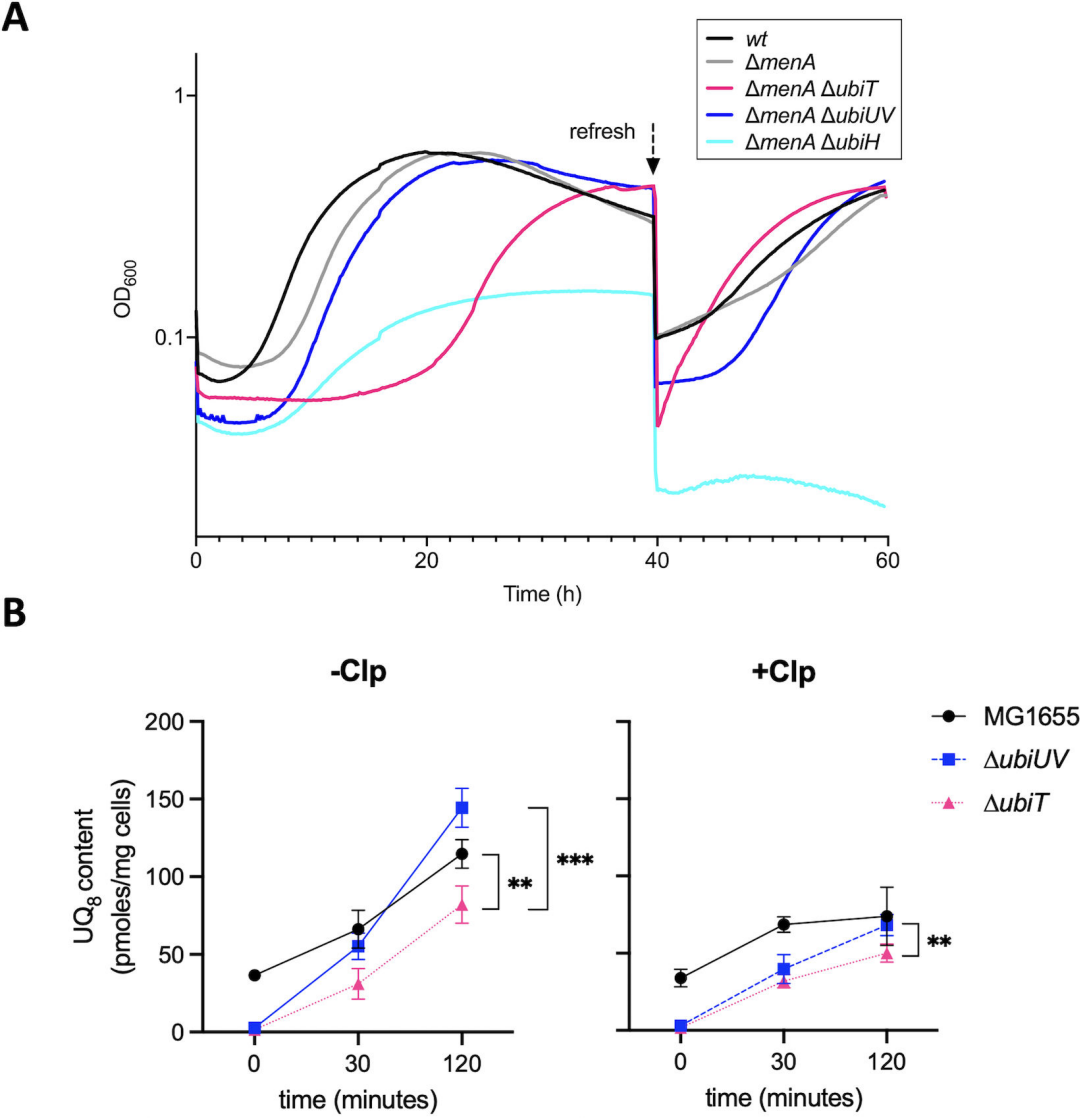
Role of *ubiUVT* in the anaerobic to aerobic transition. (**A**) *E. coli* wt and strains devoid of the MK/DMK *(*∆*menA)* and UQ aerobic (∆*ubiH*) and anaerobic (∆*ubiUV* or ∆*ubiT*) synthesis pathways were grown anaerobically in LB KNO_3_ medium, washed in M9 medium without carbon source, and resuspended in M9 succinate medium to OD_600_ = 0.02. Growth was followed aerobically at 37°C in a TECAN microplate reader in three independent experiments. At 40 hours of growth, cells were diluted 1/100 in the same medium (refresh), and growth was resumed for 20 hours more. (**B**) *E. coli* wt (MG1655), Δ*ubiUV*, and Δ*ubiT* strains were cultured anaerobically in LB medium containing NO_3_^−^ as final electron acceptor until OD ~ 1. After 20 minutes of treatment with chloramphenicol (+Clp) at 200 µg/mL or without chloramphenicol (−Clp) under anaerobic conditions, the cultures were shifted to ambient air for a 2-hour incubation. UQ_8_ content was quantified before (0 minutes) or after oxic transition (30 minutes and 120 minutes) by HPLC-ECD of lipid extracts from 1 mg of cells. Quantifications are expressed as picomole per milligram of cells (*n* = 4 biological replicates). **P < 0.01; ***P < 0.001 by unpaired Student’s *t*-test. Mean ± SD is indicated.

First, we used Δ*menA*Δ*ubiH* and Δ*menA*Δ*ubiUV* strains that only produce UQ_8_ under anaerobiosis and aerobiosis, respectively. Strains were grown in LB supplemented with NO_3_^−^ under anaerobic conditions for 24 hours, then switched to aerobic conditions with succinate as a carbon source, that is, in conditions wherein growth strictly relies on UQ_8_ (35). The wt strain showed differential efficiency in shifting from anaerobiosis to aerobiosis as compared with the ∆*menA* and Δ*menA*Δ*ubiUV* strains. Indeed, by taking the end of the lag period at the time point at which growth resumes an upward trajectory, lag periods were 2 hours for the wt and 7 hours for the ∆*menA* and the ∆*menA*∆*ubiUV* mutant strains (Fig. 8A). This suggested that UbiUVT-synthesized UQ8 does not bear a significant influence on the shift between anaerobiosis and aerobiosis. Eventually, all three strains showed the same growth rate in the exponential phase and reached the same final OD_600_ value, suggesting that the UbiIHF-synthesized UQ_8_ was activated and provided UQ_8_ in extended aerobic conditions. To confirm this hypothesis, we re-inoculated these cells into the same medium (Fig. 8A, refresh), and as expected we observed that lag periods were the same for all three strains, indicating that they had accumulated the same level of UQ_8_ since the beginning of the growth.

Surprisingly, the ∆*menA*∆*ubiH* mutant—a strain defective for the aerobic UQ_8_ synthesis pathway—was able to grow after the transition to aerobic conditions, with the same lag period as the ∆*menA* and ∆*menA*∆*ubiUV* mutant strains, that is, 7 hours. This showed that UbiUVT-synthesized UQ_8_ in the ∆*menA*∆*ubiH* allowed a shift from anaerobiosis to aerobiosis. Yet, the ∆*menA*∆*ubiH* strain showed a slower and shorter exponential phase and a lower final OD_600_ value as compared with the wt, ∆*menA*, and ∆*menA*∆*ubiUV* strains. Then, as expected, the ∆*menA*Δ*ubiH* mutant failed to resume growth on reinoculation in a fresh medium, indicating that the level of anaerobically UbiUV-synthesized UQ_8_ failed to sustain protracted aerobic growth (Fig. 8A). The fact that anaerobically synthesized UQ_8_ has a positive, yet minor effect on the anaerobic–aerobic transition, somehow contradicted the first conclusion reached when studying the ∆*menA* and ∆*menA*∆*ubiUV* mutant strains (see above). A possible explanation is that in the ∆*menA*∆*ubiUV* strain, newly synthesis of UQ_8_ by UbiIHF might be quick enough to compensate for the lack of UbiUV-synthesized UQ_8_.

UQ_8_ content was subsequently measured over a shorter time period during the transition from anaerobic to aerobic conditions in a separate experiment (Fig. 8B). For this, cultures in LB of ∆*ubiUV* or ∆*ubiT* mutants were subjected or not to chloramphenicol (Clp) treatment prior to the shift, and samples were taken at 0 minute, 30 minutes, and 120 minutes for UQ quantification. UQ_8_ level increased with time in both the wt and the ∆*ubiUV* mutant, but in the 30–120 minute period, it stopped increasing in the presence of translation inhibitor Clp. The likeliest explanation is that UQ_8_ biosynthesis is driven by UbiUV before the shift and later *de novo* synthesized by UbiIHF in aerobic conditions. This suggested that the three hydroxylases UbiI, H, and F were already present under anaerobiosis, in a standby state, waiting for O_2_ to allow hydroxylation. Importantly, this was confirmed as levels of UbiI, H, and F proteins were found to be similar in both aerobic and anaerobic conditions (Fig. S7). Also, this is consistent with the hypothesis of a very quick synthesis of UbiIHF-synthesized UQ_8_ (see above).

Second, the role of the accessory factor, UbiT, was investigated using the ∆*menA* ∆*ubiT* mutant. As described before, the ∆*menA*∆*ubiT* strain was grown first in LB with NO_3_^−^ under anaerobiosis, subsequently shifted in succinate minimal medium, and growth was monitored. A most unexpected and spectacular effect was observed as a lag period with this strain in these conditions was approximately 20 hours whereas that of the wt was approximately 2 hours (Fig. 8A). The ∆*menA*∆*ubiT* strain finally reached a final OD_600_ value similar to WT, ∆*menA*, ∆*menA*∆*ubiUV* strains at 40 hours and also resumed growth on re-inoculation at 40 hours (Fig. 8A). This highlighted a crucial role of UbiT in the anaerobic–aerobic transition phase. This result was strengthened by direct quantification of UQ_8_ synthesized with time after shifting cultures from anaerobiosis to aerobiosis (Fig. 8B). The ∆*ubiT* mutant exhibited a two-fold reduction in UQ_8_ as compared with the ∆*ubiUV* mutant after the shift. When Clp was added, the difference was much smaller. This confirmed that UbiT is necessary at the onset of aerobic UQ_8_ biosynthesis, presumably via the UbiIHF complex.

### The *yhbS* gene is not involved in UQ_8_-based metabolism

The *yhbS* gene predicted to encode an acetyltransferase lies downstream the *ubiT* gene (Fig. S8A). It was recently proposed to intervene in small noncodingRNA (sncRNA)-mediated expression control (39). Using RT-PCR, we showed that *yhbS* and *ubiT* genes share a single transcription unit (Fig. S8B). Using YhbS-SPA tag protein, we observed that YhbS protein synthesis takes place both under aerobiosis and anaerobiosis. The level of YhbS-SPA protein appears slightly higher in −O_2_, and this induction seems to be lost in the ∆*fnr* mutant, as expected if *yhbS* and *ubiT* genes are co-expressed and co-regulated by Fnr (Fig. S8C). The ∆*yhbS* mutant shows no defect in NO_3_^−^ respiratory capacity, and no aggravating effect was observed on combining ∆*yhbS* and ∆*menA* mutations (Fig. S8D). Last, we carried out shift experiments, from −O_2_ to +O_2_, as described above for *ubiT* and failed to identify any defect in the ∆*yhbS* mutant (not shown). Altogether with previous assays failing to reveal a defect in UQ_8_ levels in anaerobiosis in the ∆*yhbS* mutant (3), these results allowed us to rule out a role of YhbS in UQ_8_ synthesis.

## Discussion

UQ is an essential component of electron transfer chains and of respiratory metabolism. For decades, the dogma has been that UQ was exclusively used for aerobic respiratory metabolism, whereas DMK/MK was used for electron transfer in anaerobic respiratory chains. Following our recent discovery that UQ is also synthesized under anaerobiosis, which contradicted the above assumption (3), the present study identified two versatile anaerobic physiological processes that rely on the anaerobic UQ biosynthesis pathway, namely NO_3_^−^ respiration and uracil biosynthesis. Moreover, we provide clear evidence that UbiUV catalyzes hydroxylation steps independently from O_2_. Last, UbiT was found to play a key role in both anaerobiosis and aerobiosis conditions, allowing a smooth transition between the two conditions. Overall, this analysis uncovers a new facet of the strategy used by *E. coli* to adapt to changes in O_2_ levels and respiratory conditions. This is of particular interest in the context of gut microbiota studies, as changes in O_2_ level and in respiratory electron acceptors are key factors that the host uses to select the type of flora present through the different sections of the intestine (40).

UbiUV-mediated UQ synthesis takes place under anaerobiosis. Here we showed that this is made possible by Fnr-mediated activation of expression of the *ubiUV* operon that takes place from microaerobiosis (0.1% O_2_) to anaerobiosis. In contrast, expression of the *ubiT* gene is more versatile with two promoters, one under Fnr control, allowing UbiT synthesis under microaerobiosis and anaerobiosis, simultaneously with UbiUV, and the second constitutive one, insuring expression in aerobiosis. This genetic regulation is consistent with the presence of UbiT proteins under both aerobic and anaerobic conditions. Such a versatile expression meets with other evidence we collected, which together pave the way to an important role of UbiT in the anaerobiosis to aerobiosis transition: (i) UbiT is required for insuring continuous UQ synthesis on shifting from anaerobiosis to aerobiosis, (ii) *ubiT* was found to compensate for the lack of *ubiJ* in conditions where high dosage of *ubiUV* genes suppressed absence of *ubiIHF* under aerobiosis, and (iii) UbiIHF enzymes are present in anaerobiosis but not active as one would expect for O_2_-dependent hydroxylases. This indicates that the O_2_-dependent pathway is in a standby mode in anaerobic conditions, waiting only for the presence of O_2_ to activate the O_2_-dependent hydroxylases and produce UQ, as proposed previously (41). This is also consistent with the fact that UbiUV synthesis is strictly controlled at the transcriptional level, whereas expression of *ubiIHF* is constitutive. Altogether, this leads us to propose that UbiT and UbiJ are required for the formation of two related but distinct metabolons, respectively, an anaerobic one containing UbiUV and an aerobic one containing UbiIHF. Besides, both UbiJ and UbiT are likely to bind UQ biosynthetic intermediates via their SCP2 domain, thereby providing the substrates to UbiUV and UbiIHF (9, 42).

UbiUV catalyzes hydroxylation of the benzene ring in the absence of O_2_. Moreover, our results show that they can substitute for aerobic hydroxylases UbiIHF in the presence of O_2_, but that they still catalyze the hydroxylation without relying on O_2_ in this condition. This raises the question of the source of the O atom under anaerobiosis. Previous analysis on RhlA, a member of the U32 protein family to which UbiU and V belong, indicated that prephenate, an intermediate within the aromatic amino acid biosynthesis pathway, could act as an O donor (11). Our ongoing studies aim at investigating such a possibility in the case of anaerobic UQ biosynthesis. [Fe–S] clusters seem to play a role in the process since *isc* mutants devoid of anaerobic [Fe–S] biogenesis machinery and UbiU variant lacking [Fe–S] cluster fail to produce UQ. The simplest hypothesis is that [Fe–S] clusters are transferring electrons from the O source to a terminal reductase, both to be identified.

UbiUVT-synthesized UQ has a significant contribution to growth in anaerobiosis and microaerobiosis (0.1% O_2_). Indeed, we found that UbiUVT-synthesized UQ are key for NO_3_^−^ respiration in the absence of DMK, in agreement with early biochemical work on formate-nitrate reductase (37) and with our previous study reporting that *P. aeruginosa-* denitrifying activity depends on UbiUVT-synthesized UQ (42). Moreover, we observed that the anaerobically synthesized UQ greatly contributes to uracil synthesis. This was unexpected as uracil synthesis was reported to depend mainly on the oxidation of (S)-dihydroorotate to orotate with fumarate as a hydrogen acceptor and DMK/MK as an electron carrier (37). Our present physiological studies demonstrate that the anaerobically produced UQ can fully compensate for the DMK/MK loss, likely through an as yet unknown reductase since UQ is too electropositive to be a frdABCD substrate (43). Last, UQ could be used as an electron sink to other catabolic processes taking place in both aerobiosis and anaerobiosis such as heme biosynthesis, wherein the HemG enzyme utilizes UQ or MK for the conversion of protoporphyrinogen IX into protoporphyrin IX (44).

The contribution of anaerobically synthesized UQ for *E. coli* multiplication in the gut appeared as marginal. This implies that either absence of UV-synthesized UQ was masked by MK/DMK synthesis or anaerobic UQ-dependent processes such as NO_3_^−^ respiration or uracil biosynthesis is dispensable. Clearly, the first possibility is the likeliest given the paramount importance of anaerobic respiration for *E. coli* multiplication in the gut (45, 46), as nicely confirmed by the drastically altered multiplication of MK/DMK-deficient cells (Fig. 7). This is of particular interest as the presence and nature of respiratory electron acceptors were proposed to be drivers of bacterial community composition in the different regions of the intestine (40). Likewise, the relatively high O_2_ level in the duodenum, of NO_3_^−^ in ilium, and hypoxia in the cecum were proposed to be causal of the different flora hosted in these regions in a healthy host. Strategies used by *E. coli* to live in such different respiratory and fermentative conditions are therefore key aspects of its adaptation to the host. In this context, it is important to understand the mechanism underlying the switch from O_2_-rich to NO_3_^−^-rich and/or hypoxic compartments, and the present study highlights the added value of having overlapping systems permitting a smooth shift from anaerobic NO_3_^−^ to aerobic respiration.

## SUPPLEMENTAL MATERIAL

Figure S1 mbio.03298-22-s0001.tif UQ biosynthetic pathway.

Figure S2 mbio.03298-22-s0002.tif UbiV-SPA amounts.

Figure S3 mbio.03298-22-s0003.tif Quinone content in *isc* and *suf* mutants.

Figure S4 mbio.03298-22-s0004.tif UbiT-SPA amounts.

Figure S5 mbio.03298-22-s0005.tif UQ content in strains used for colonization experiments.

Figure S6 mbio.03298-22-s0006.tif cfu counts of colonization experiments.

Figure S7 mbio.03298-22-s0007.tif Amounts of UbiIHF proteins.

Figure S8 mbio.03298-22-s0008.tif *ubiT* is in operon with the unknown function *yhbS* gene.

ASM does not own the copyrights to Supplemental Material that may be linked to, or accessed through, an article. The authors have granted ASM a non-exclusive, world-wide license to publish the Supplemental Material files. Please contact the corresponding author directly for reuse.
