# Supplementary figures and images for: Role of the Escherichia coli ubiquinone-synthesizing UbiUVT pathway in adaptation to changing respiratory conditions
Source: mBio. 2023 Jun 7;14(4):e03298-22. doi: 10.1128/mbio.03298-22 (PMC10470549; doi:10.1128/mbio.03298-22)

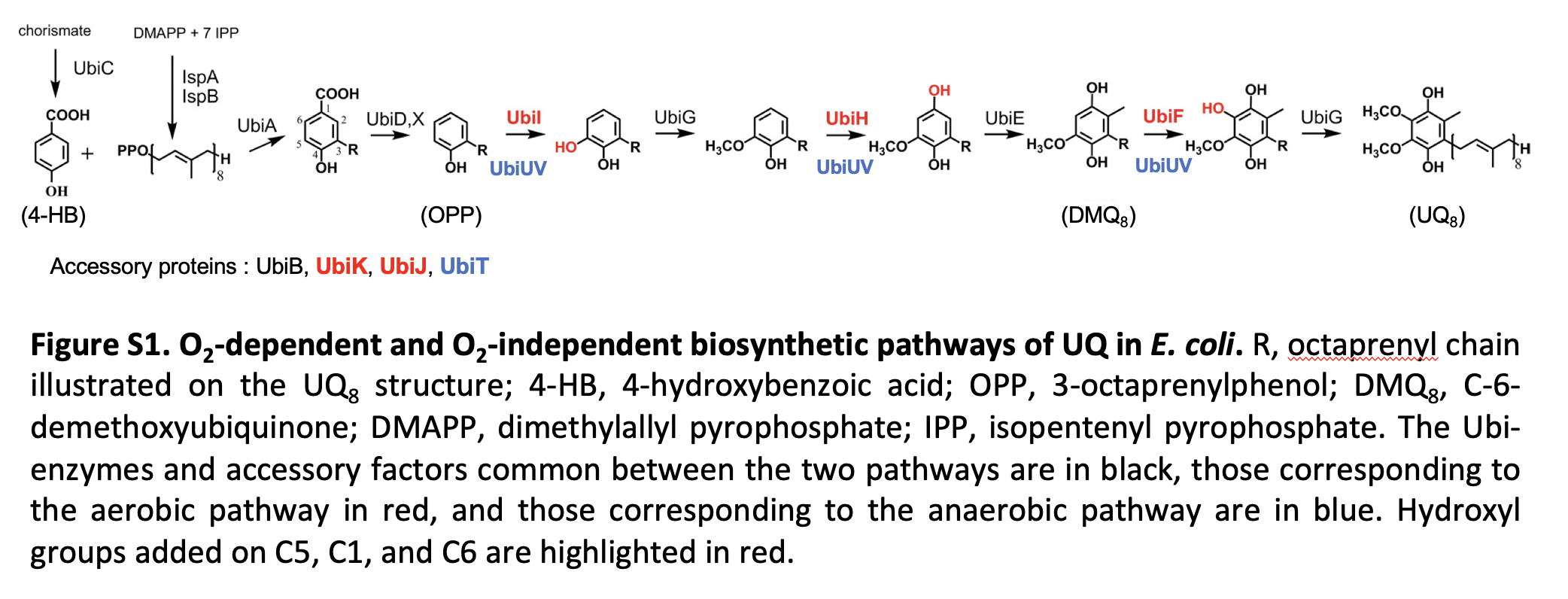

Supplement: Figure S1 — UQ biosynthetic pathway. [file mbio.03298-22-s0001.tif]

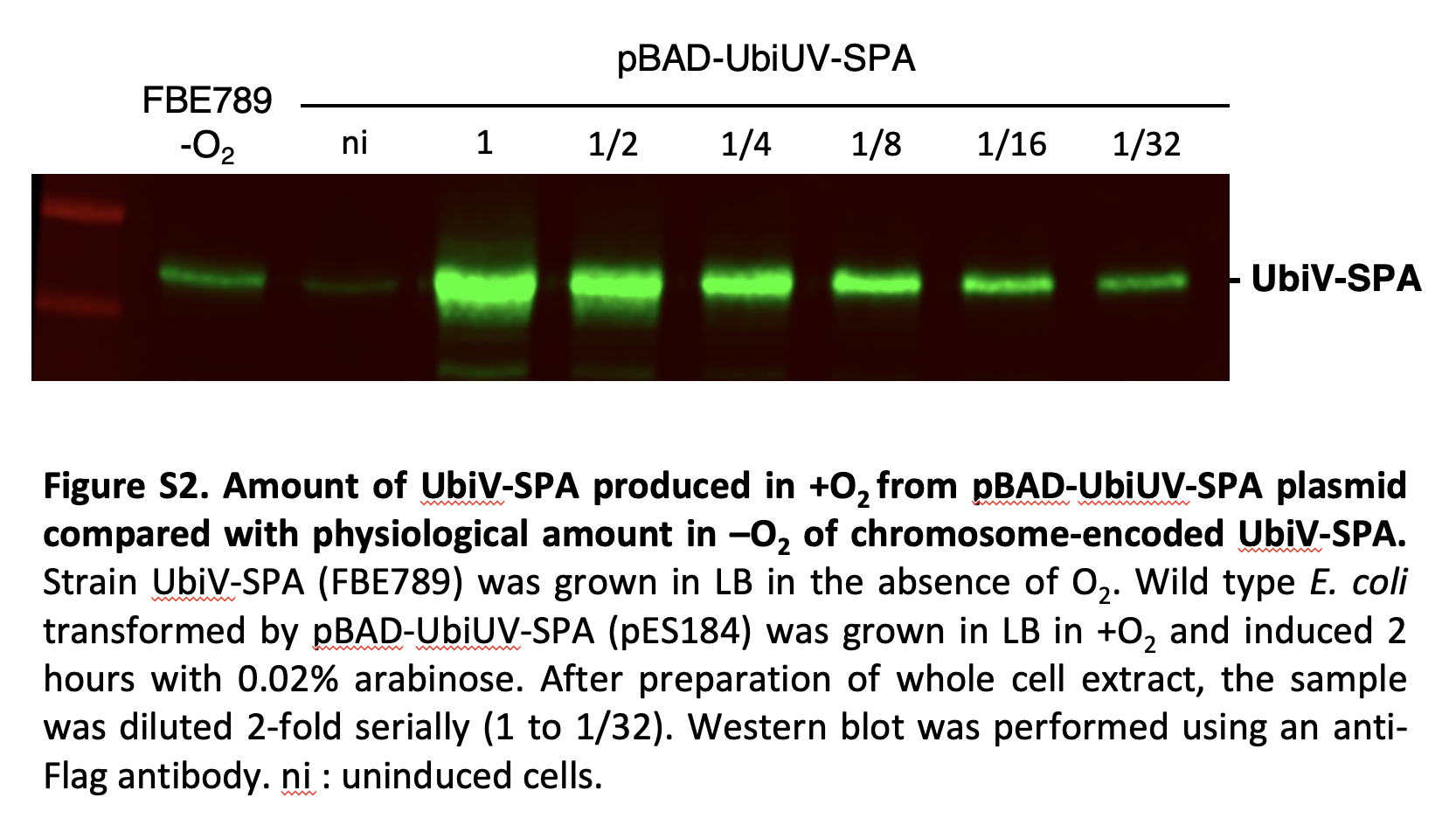

Supplement: Figure S2 — UbiV-SPA amounts. [file mbio.03298-22-s0002.tif]

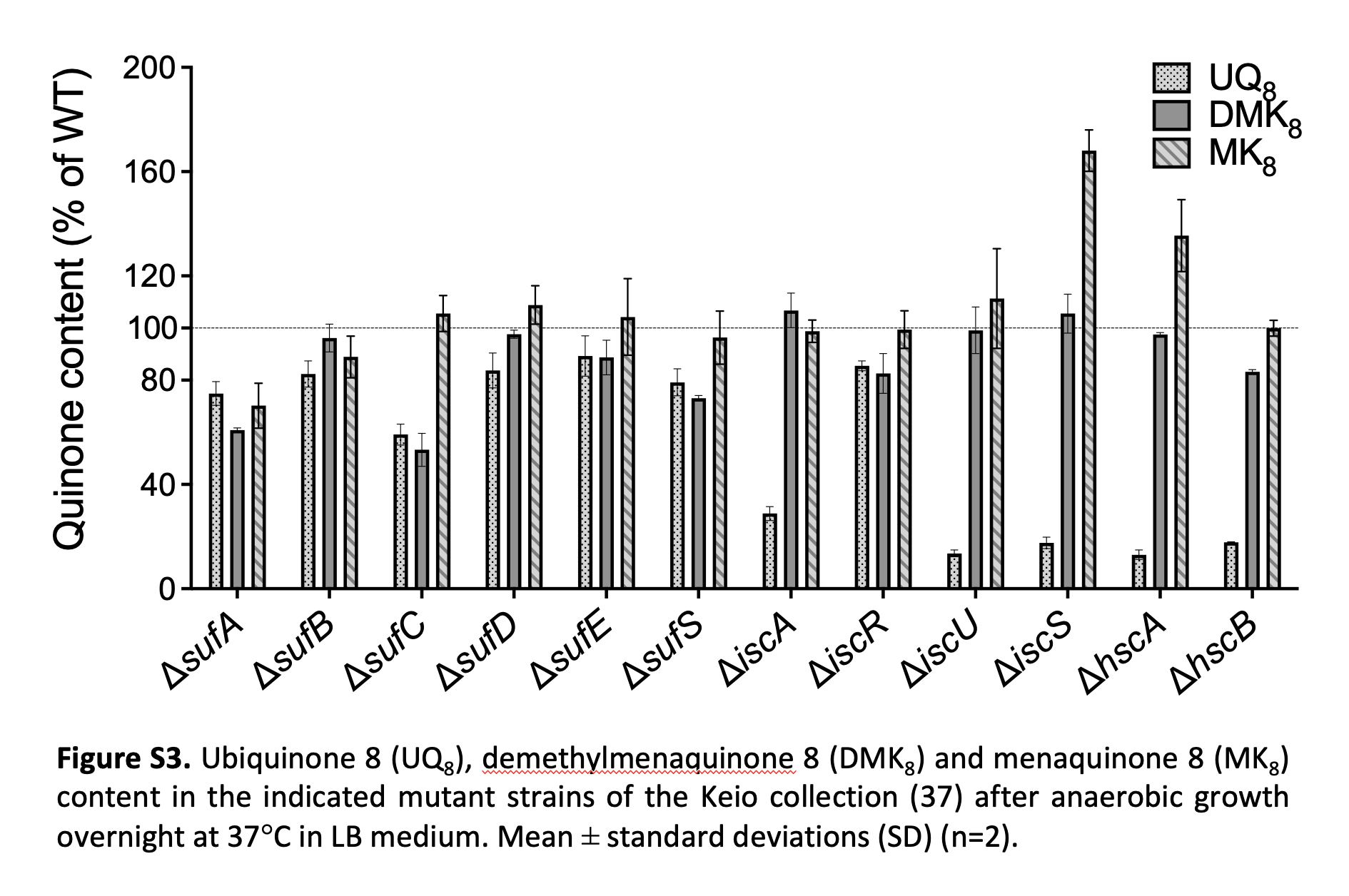

Supplement: Figure S3 — Quinone content in isc and suf mutants. [file mbio.03298-22-s0003.tif]

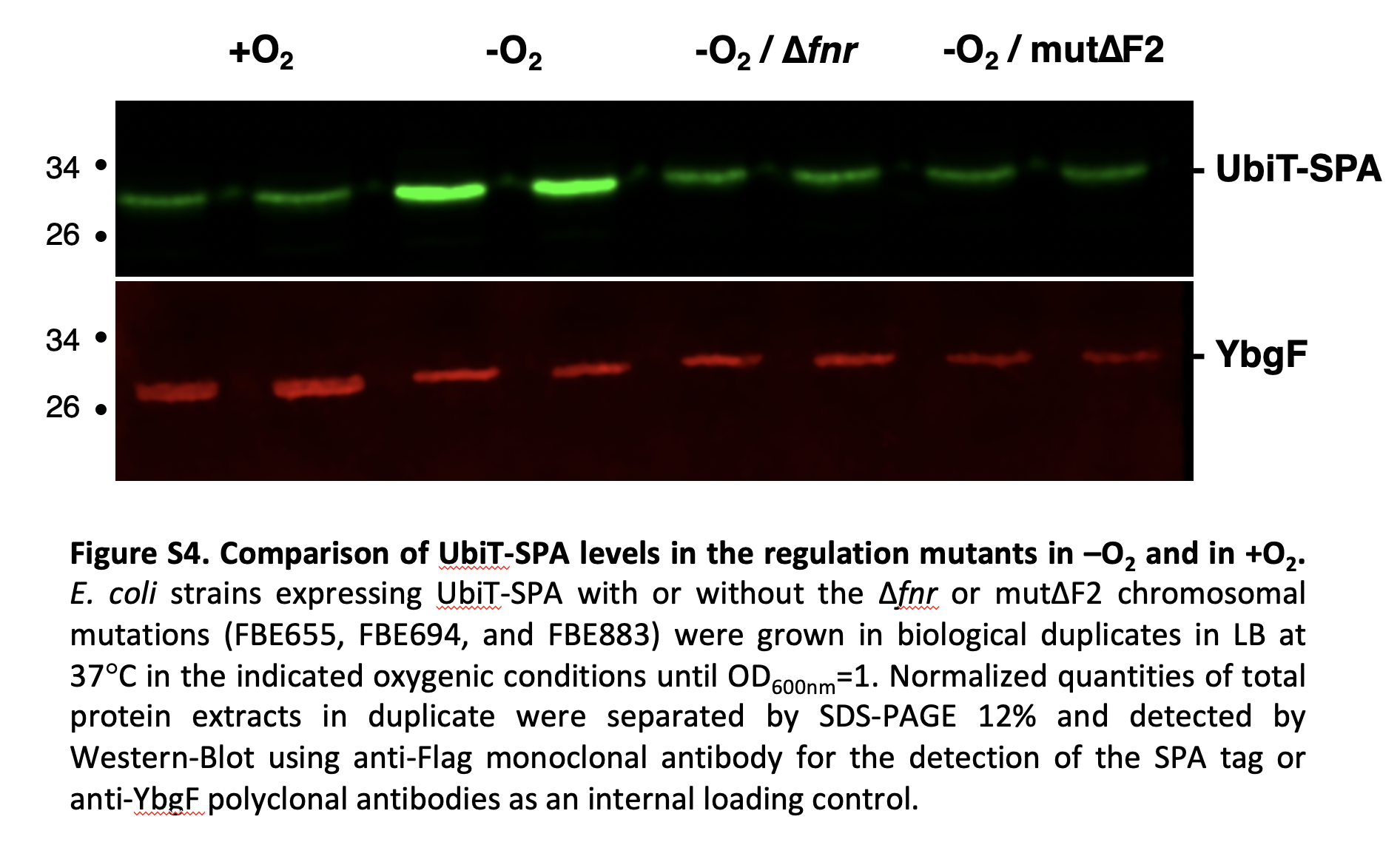

Supplement: Figure S4 — UbiT-SPA amounts. [file mbio.03298-22-s0004.tif]

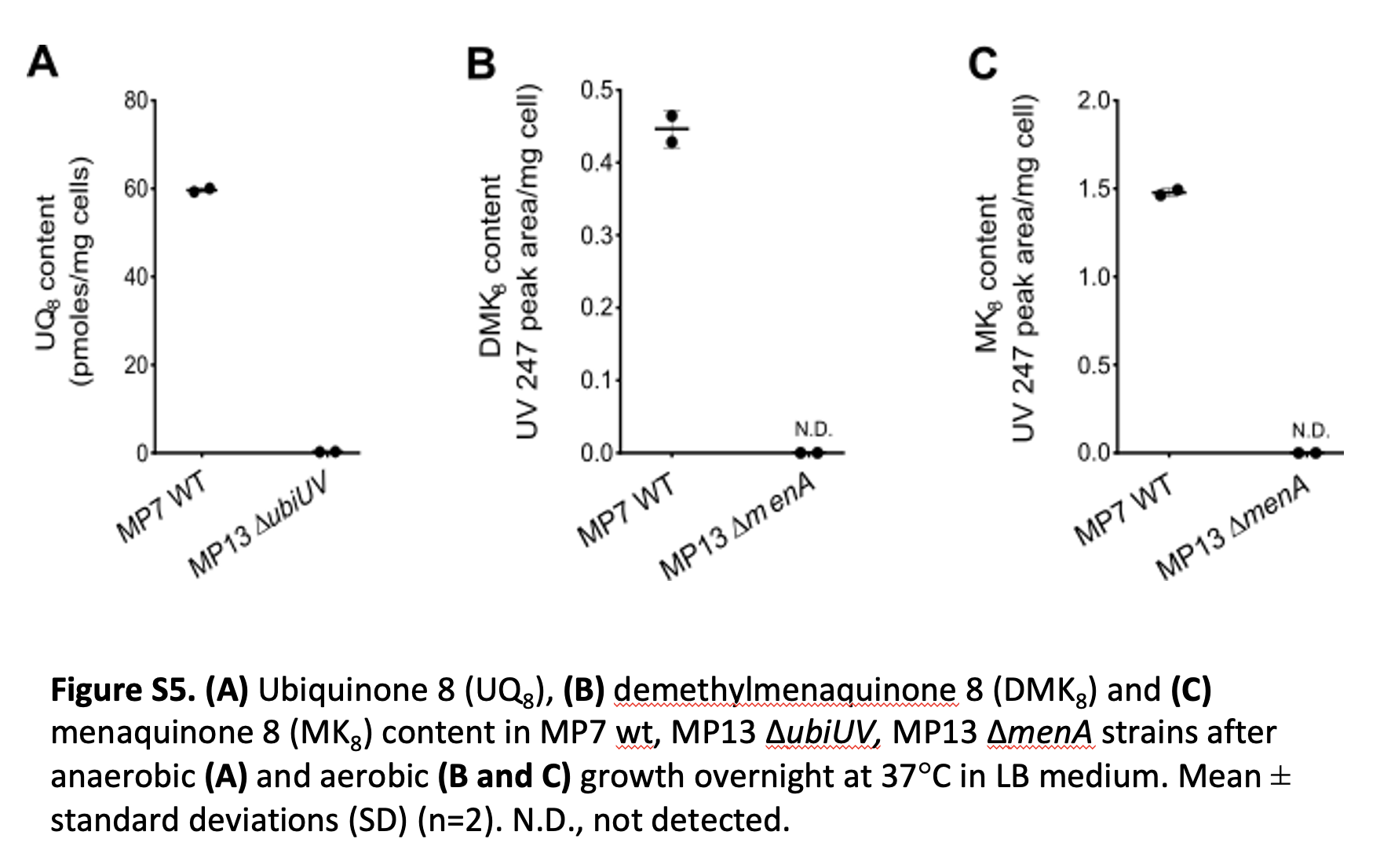

Supplement: Figure S5 — UQ content in strains used for colonization experiments. [file mbio.03298-22-s0005.tif]

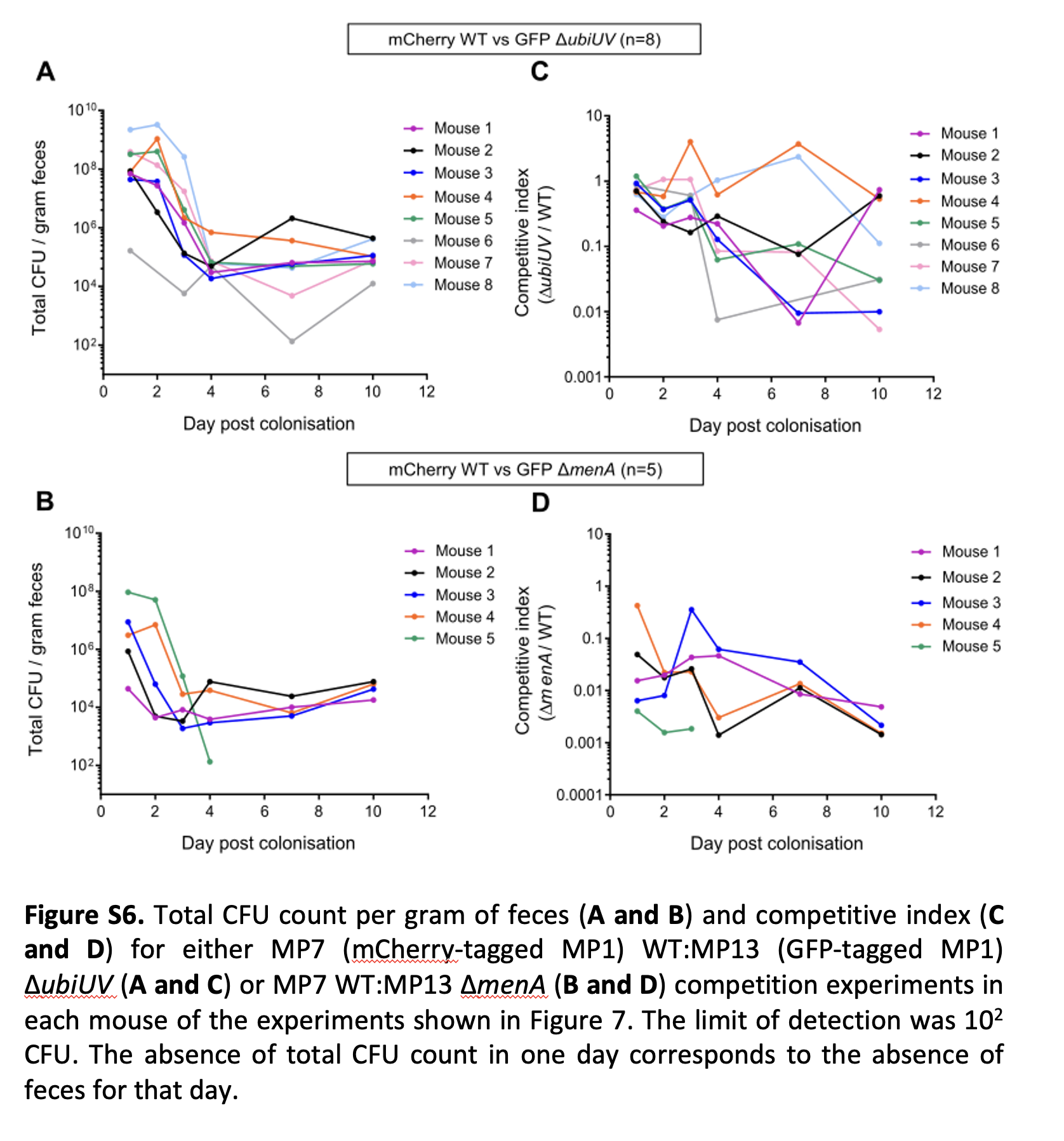

Supplement: Figure S6 — cfu counts of colonization experiments. [file mbio.03298-22-s0006.tif]

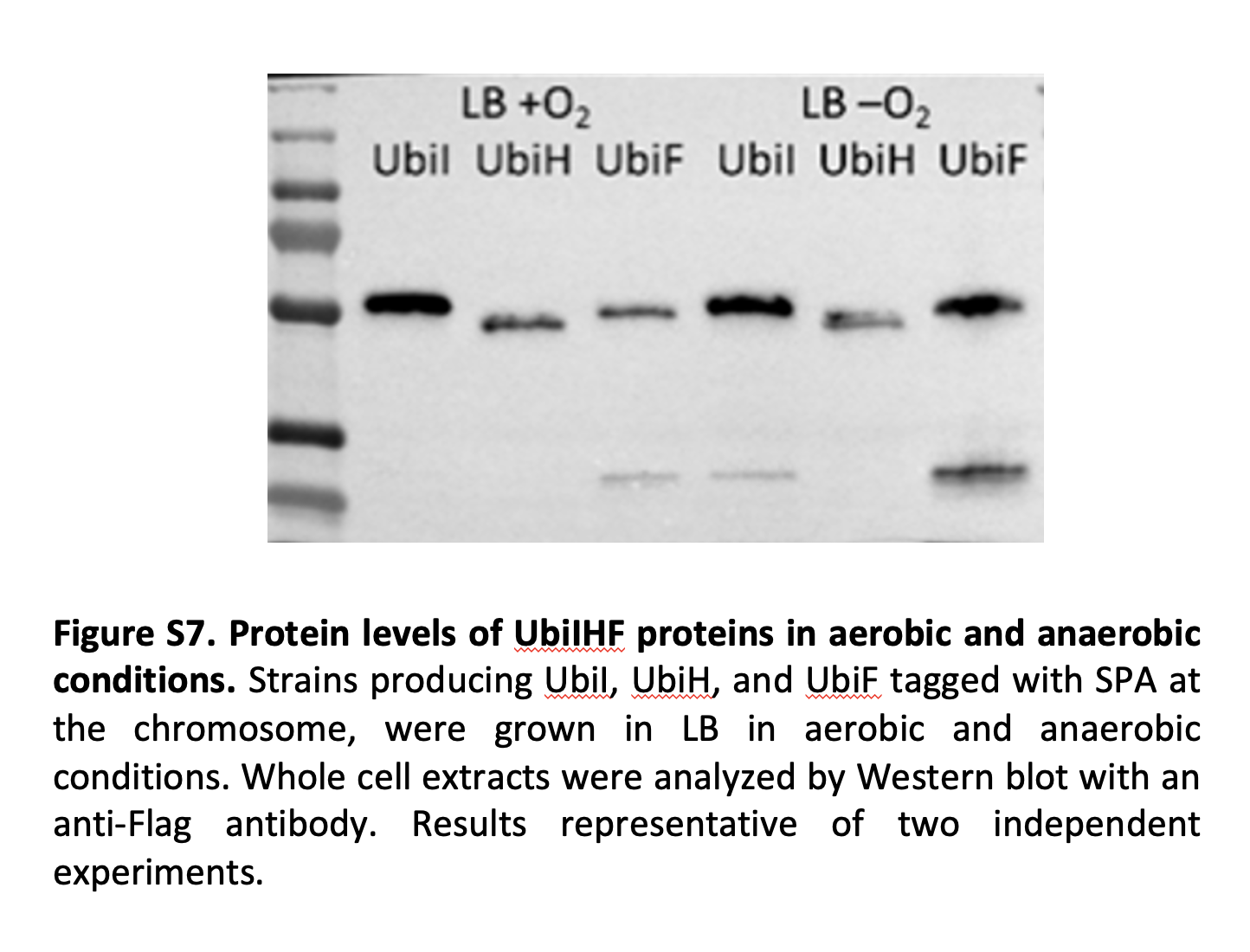

Supplement: Figure S7 — Amounts of UbiIHF proteins. [file mbio.03298-22-s0007.tif]

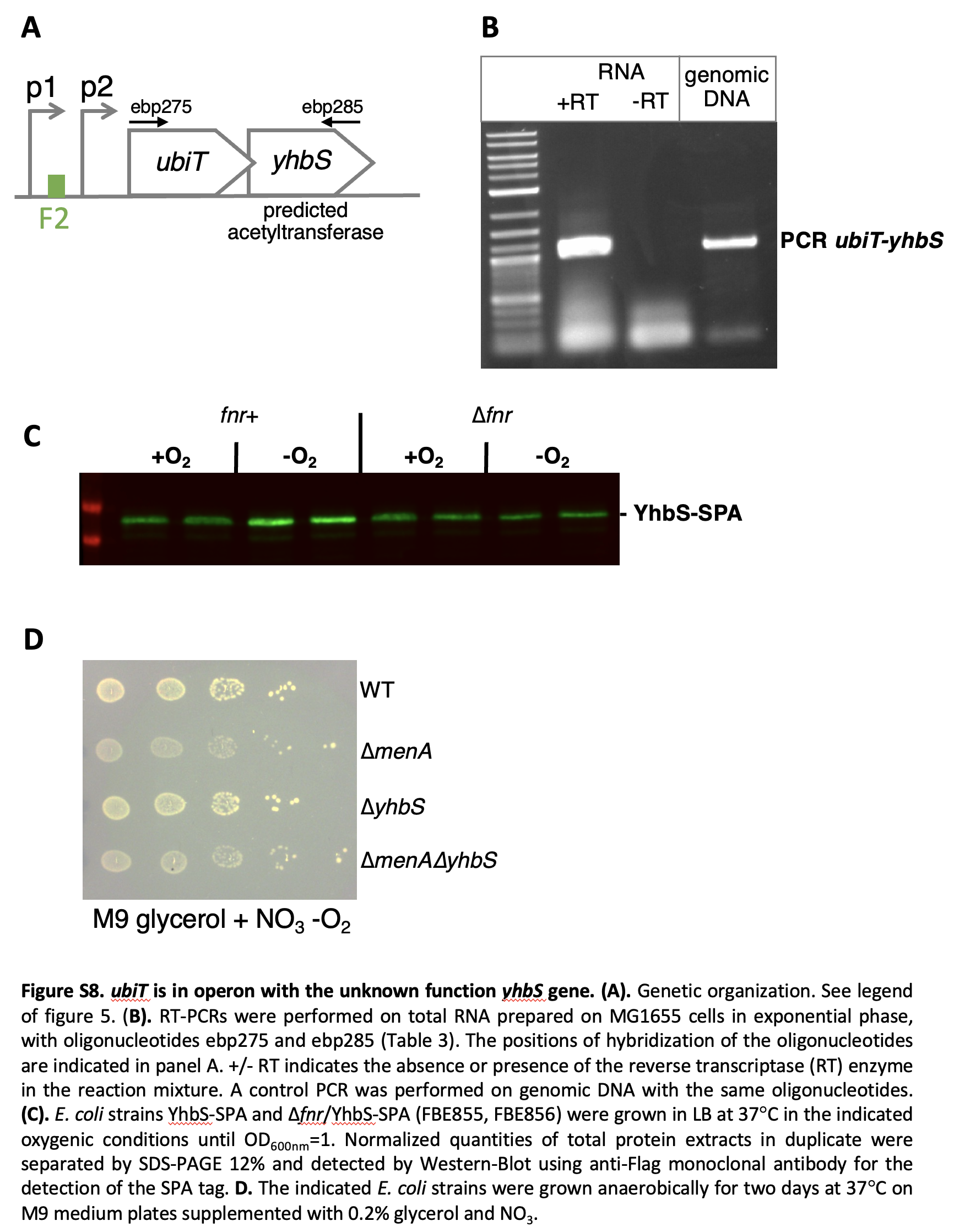

Supplement: Figure S8 — ubiT is in operon with the unknown function yhbS gene. [file mbio.03298-22-s0008.tif]
